# Parity modifies the effect of genetic variants associated with gestational duration and birth weight

**DOI:** 10.1101/2025.06.17.25329777

**Published:** 2025-06-17

**Authors:** Karin Ytterberg, Hedvig Sundelin, Julius Juodakis, Marc Vaudel, Elizabeth C. Corfield, Ole A. Andreassen, Per Magnus, Alexandra Havdahl, Staffan Nilsson, Pål R. Njolstad, Stefan Johansson, Bo Jacobsson, Pol Solé-Navais

**Affiliations:** 1 Department of Obstetrics and Gynecology, Institute of Clinical Sciences, Sahlgrenska Academy, University of Gothenburg, Gothenburg, Sweden; 2 Mohn Center for Diabetes Precision Medicine, Department of Clinical Science, University of Bergen, Bergen, Norway; 3 Department of Genetics and Bioinformatics, Health Data and Digitalization, Norwegian Institute of Public Health, Oslo, Norway; 4 PsychGen Centre for Genetic Epidemiology and Mental Health, Norwegian Institute of Public Health, Oslo, Norway; 5 Research Department, Lovisenberg Diaconal Hospital, Oslo, Norway; 6 MRC Integrative Epidemiology Unit, Population Health Sciences, Bristol Medical School, University of Bristol, Bristol, UK; 7 NORMENT Centre, University of Oslo, Oslo, Norway; 8 Institute of Clinical Medicine, Faculty of Medicine, University of Oslo, Oslo, Norway; 9 Centre for Fertility and Health, Norwegian Institute of Public Health, Oslo, Norway; 10 PROMENTA Research Center, Department of Psychology, University of Oslo, Oslo, Norway; 11 Department of Pediatrics and Adolescents, Haukeland University Hospital, Bergen, Norway; 12 Department of Medical Genetics, Haukeland University Hospital, Bergen, Norway; 13 Department of Genetics and Bioinformatics, Division of Health Data and Digitalization, Norwegian Institute of Public Health, Oslo, Norway; 14 Wallenberg Laboratory/ Department of Molecular and Clinical Medicine, Institute of Medicine, Sahlgrenska Academy, University of Gothenburg, Gothenburg, Sweden.

**Keywords:** gestational duration, birth weight, parity, nulliparity, preterm birth, Gene × environment interaction, The Norwegian Mother, Father and Child Cohort Study (MoBa), Medical Birth Registry of Norway (MBRN)

## Abstract

Gestational duration and birth weight are linked to both short- and long-term adverse health outcomes in mothers and their offspring. Previous genome-wide association studies on these pregnancy outcomes have been successful but have overlooked the number of a mother’s previous pregnancies. In this study, we explored if parity (the number of children a mother has previously delivered) modifies the maternal or foetal genetic effect of gestational duration and birth weight (gene × parity interactions) using data from the Norwegian Mother, Father, and Child Cohort Study in up to 58,528 mothers and their offspring. Potential genetic effect differences were investigated by: (1) performing parity-stratified genome-wide association studies, (2) testing SNP × parity interactions, (3) testing the interaction between a polygenic prediction of the traits and parity, and (4) assessing the genetic correlation within each outcome across parity. For both phenotypes, we identified shared and distinct loci for each outcome in terms both of genetic region and number in the parity-stratified genome-wide association studies, and the genetic correlation by parity deviated from one for all outcomes. The strongest evidence of genetic modification effect by parity was found for gestational duration in the maternal genome where genetic effects were stronger in the first pregnancy compared to later pregnancies. For instance, the polygenic prediction of the maternal genome on gestational duration had a significant interaction with parity (p-value interaction = 5 × 10^−5^). The results for birth weight were more uncertain, suggesting that the identified gene × parity interactions is largely limited to gestational duration. In conclusion, our study reveals that parity modifies the genetic effects on gestational duration and highlights the relevance of considering parity in genetic studies on pregnancy outcomes.

## Introduction

The duration of gestation and offspring’s birth weight are linked to adverse short- and long-term health outcomes in both the mother and the neonate. For instance, preterm birth (before 37 weeks of gestation) is the leading cause of death in neonates and children under five years of age(1). Gestational duration and birth weight are complex traits with polygenic architectures; previous genome-wide association studies (GWASs) have identified multiple genetic loci associated with these birth outcomes in both the maternal and foetal genomes (2–6). While the maternal genome has a larger role in shaping the duration of gestation compared to the foetal one, it’s the opposite for birth weight; the foetal genome contributes more to its genetic architecture than the maternal one. Overall, 30 and 250 loci have been identified to date for gestational duration and birth weight, respectively. However, these GWASs have overlooked information on the number of previous pregnancies of a mother.

Parity, the number of children a mother has previously delivered, is an important clinical factor. Today, we know that gestational duration is shortest in the first pregnancy (and has higher variance) (7), and that birth weight (8, 9) also tends to be lower in the first pregnancy compared to later ones. The immune system (10, 11) and uterine size (12) differ by parity and may potentially contribute to the differences observed in the mean and distributions of these pregnancy outcomes. These mechanisms could also play a role in modulating the genetic effects of the maternal and foetal genomes on the duration of gestation (7) and birth weight, but this has been largely ignored in GWASs performed to date.

The classical approach to testing gene × environment interaction effects is to investigate each genetic variant separately (13–15), which has a high multiple testing burden. To increase power, different polygenic approaches have been proposed, including investigating potential heritability differences or estimating genetic correlations between the different environmental groups (16–18). An alternative is to assess whether the environmental factor interacts with the cumulative effect of thousands of genetic variants across the genome, represented as a polygenic score (18–21).

Therefore, in this study we investigated whether parity modifies the maternal or foetal genetic effects on gestational duration and birth weight in up to 58,000 mothers/ children. We explored parity-stratified GWASs, single SNP × parity interactions, if the polygenic prediction of any outcome interacts with parity, and lastly the genetic correlation between parities. Our results highlight that parity modifies the genetic effect on gestational duration and birth weight in both the maternal and foetal genome.

## Methods

### Study cohort

The Norwegian Mother, Father, and Child Cohort Study (MoBa) is a population-based pregnancy cohort conducted by the Norwegian Institute of Public Health. Participants were recruited from all over Norway from 1999 and 2008. The women consented to participation in 41% of the pregnancies. The cohort includes approximately 114,500 children, 95,200 mothers and 75,200 fathers (22). Blood samples were obtained from both parents during pregnancy and from mothers and children (umbilical cord) at birth (23). The current study is based on version 12 of the quality-assured data files released for research in 2019. MoBa participants responded to questionnaires during and after the pregnancy and have been linked to Norwegian registries. The data used in this study also includes data from the Medical Birth Registry of Norway (MBRN; a national health registry containing information about all births in Norway from 1967 onwards) (24). In this study, genotyping data from 207,569 unique individuals of European ancestry (76,577 children, 53,358 fathers, and 77,634 mothers) and 6,981,748 SNPs that passed the MoBaPsychGen pipeline were analysed (25). Genotyping, pre-imputation quality control (QC), phasing, imputation, and post-imputation QC have previously been described in full (25).

### Sample selection

To be included in this study participants needed to be registered in both MoBa and MBRN. For mothers with several pregnancies included in this set of genotyped MoBa samples (n = 9,923), one random pregnancy per mother was included when analysing the maternal genome to ensure independent samples for the analysis. This is important for the parity-stratified analyses as it prevents any single mother from being represented in multiple parity groups. For the analysis using the maternal or foetal genome, individuals were restricted based on relatedness; not allowed to be full siblings (king-cutoff 0.1327) (26).

For the two phenotypes, the analysis was restricted to singleton live births conceived without the use of assisted reproductive technology. Records were excluded if the sex assigned at birth of the child was unknown, the child was born with malformations, or if there was an early perinatal death. For definitions and specific criteria within each phenotype and the exposure parity see below.

#### Gestational duration

Gestational duration is based on the records from MBRN, it is measured in days and based on ultrasound estimation in the mid second trimester. However, if ultrasound is not available the gestational length is calculated from the last menstrual period (in this cohort more than 98% of the pregnancies were estimated with ultrasound). The analysis was restricted to spontaneous births, with a gestational duration ranging between 154 to 308 days. Children dying within one year from birth were not included.

#### Birth weight

The offsprings birth weight (in gram precision) was obtained from the MBRN. The analyses of the birth weight were limited to gestational durations between 259 and 300 days. Children with a birth weight within ± 5 standard deviations from the mean birth weight were included. The birth weight was transformed to z-scores for all analyses using the mean and standard deviation from the study population.

#### Parity

Parity is defined as the number of times a woman has previously given birth to a child, live or stillborn. In MBRN parity is determined as the greater of “Previous births reported by the mother” and “Previous births recorded in the MBRN”. Previous births were registered in MBRN from week 16 (112 days) between 1967 and 2001, and from 2002 onwards, they were registered from week 12 (84 days). The counting of parity is zero-based, meaning that the first pregnancy is parity zero (nulliparous) and so on. Mothers with uncertain parity were removed to avoid potential errors in parity: mothers who gave birth to more than one child with the same parity, and cases where the parity did not correspond to the order of delivery (for example, a parity zero occurred after a parity one).

### Genome-wide association studies

The GWASs were performed using REGENIE (v3.2.5) (27) with analyses consisted of three groups: whole sample, first pregnancy, and later pregnancies for the two phenotypes (gestational duration and birth weight) when analysing either the maternal or foetal genome. This resulted in a total of 12 GWASs (Table 1). For gestational duration, the regression models were adjusted for maternal age, foetal sex assigned at birth, genotyping chip and the ten first genotype principal components. For birth weight the GWASs were adjusted for foetal sex assigned at birth, genotyping chip, gestational duration, and the ten first genotype principal components. Top lead genetic variants were those with an association p-value below the genome-wide significance threshold (5 × 10^−8^) with a radius of 1.5 × 10^6^ base pairs.

### Heritability and genetic correlation

SNP heritability was estimated with LD score regression (LDSC) (v 1.0.1) (28), using pre-computed LD Scores from 1000 Genomes European data and GWAS summary statistics as input. The genetic correlation between first pregnancies and later pregnancies were also estimated using LDSC (29) for the two phenotypes and the maternal and foetal genome.

### Genome-wide gene × environment interaction study

A genome-wide gene × environment interaction study on the whole sample for each phenotype and genome was conducted with REGENIE (v3.2.5) as a linear regression and included parity, SNP and the interaction SNP × parity terms. The model also adjusted for the same covariates as in the GWASs explained above.

### Polygenic scores and prediction of pregnancy outcomes using random forest models

The goal of the random forest genetic score was to reflect the genetic effects on the two phenotypes based on genetic variants reaching genome-wide significance in previous meta-analyses (2–6). To prevent splitting the data into entirely separate training and testing sets, which would reduce the sample size for testing the gene × parity interaction, out-of-bag predictions were applied on the random forest models (30). This approach allowed us to use the full data both when constructing the genetic prediction and when assessing if parity interacts with the genetic prediction (top variants across the entire genome). Moreover, a benefit of using random forest is that it allows to capture non-linear associations.

Random forest models were created to predict the two phenotypes using maternal or foetal hard-coded genotypes (coded as 0, 1, 2) of the genetic variants previously reaching genome-wide significance in the GWASs (2–6) for each outcome. Each phenotype (gestational duration and birth weight z-score) was residualised as the mean plus the individuals’ residual from a linear model adjusting for the same covariates as in the GWASs mentioned above: maternal age (for gestational duration model), gestational duration (for birth weight model), foetal sex assigned at birth, genotyping chip and the ten first genotype principal components (for both). The prediction accuracy of the random forest models represents the genetic predictability of the phenotype and was estimated as $r_{\mathrm{out}-\mathrm{of}-\mathrm{bag}}^{2}=1-\frac{\mathrm{Mean}\;\mathrm{square}\;\mathrm{error}\;}{\sigma^{2}(\text{Residualized}\;\mathrm{Gestatonal}\;\mathrm{duration})}$. From the random forest models, we obtained genetically predicted residualised gestational durations/birth weight z-scores, we will refer to these as random forest genetic scores. Since random forests have a random component (each decision tree in the forest is created using a random subset of the dataset and each node in a tree is created using a random subset of variables), a mean random forest genetic score was created for each individual based on 10 000 out-of-bag predictions with the tuned hyperparameters. The mean random forest genetic score was used to test for gene × parity interaction on each outcome in a linear model for the same samples used to create the random forest model (Phenotype = β_0_ + β_1_*random forest genetic score + β_2_*parity + β_3_*random forest genetic score*parity, the random forest genetic score was centered to have a mean of zero). Furthermore, two similar random forest models of the residualised phenotypes for each parity group were created separately to estimate the genetic predictability by parity for each outcome. The whole sample, and split for first pregnancy and later pregnancies, were also used to construct random forest models not only using genetic features but also including the abovementioned covariates (i.e., the outcome was non-residualised). All models created for each phenotype are summarized in Table 2. All models were built in R using the ranger package (31). For the performance of the random forest, hyperparameters were tuned (Supplementary Method). To estimate how well each model performs in terms of genetic predictability, a 95 % credible interval was generated from 10 000 r^2^ on out-of-bag predictions from each model, using the tuned hyperparameters.

### SNP proxies for missing variants

Some of the previously identified variants associated with gestational duration or birth weight did not pass quality control or were not part of the imputation panel. To retain the maximum information, variants present in our genotype data within +/− 550 000 base pairs of the missing top SNP were extracted. The linkage disequilibrium pattern of each genomic region was then explored using the LDassoc Tool from LDlink (32). If a variant within the genomic region had an r^2^ > 0.3 to the missing top SNP, this SNP was used as a proxy. When several variants reached an r^2^ > 0.3 with the missing variant, the variant with the largest r^2^ was selected. After searching for proxies there were still some top SNP missing (Table 3).

### Expression QTL loop-ups

To connect lead SNPs to potential causal genes, we looked up each of them in the eQTL Catalogue (33). This resource provides a uniformly processed set of expression and splice QTLs in 127 datasets from a multitude of tissues and cell types. More specifically, we searched whether the lead SNPs we have identified were part of fine-mapped sets, and if so, which were the potential target genes and in which tissues/ cell types.

### Ethical approval

Informed consent was obtained from the study participants. The study protocol was approved by the administrative board of the Norwegian Mother, Father and Child Cohort Study which is led by the Norwegian Institute of Public Health. MoBa is regulated by the Norwegian Health Registry Act. The establishment of MoBa and initial data collection was based on a license from the Norwegian Data Protection Agency and approval from The Regional Committee for Medical Research Ethics. The current study was approved by The Norwegian Regional Committee for Medical and Health Research Ethics (#2012/67 and # 2015/2425) and by The Swedish Ethical Review Authority (Dnr #2025–03908-02).

### Data and code availability

Due to sensitive information, access to raw data requires an application to the administrative board of the Norwegian Mother, Father and Child Cohort Study, conditioned on an approved ethical application. Summary statistics from the GWASs created in this project (maternal and foetal GWASs on gestational duration and birth weight in whole study population and stratified by parity) will be available upon publication.

Analyses were executed with Ubuntu 20.04.4, Long Term Support, and analysis workflow was managed using Snakemake (34), in a defined Conda environments (35). The code will be available upon publication.

## Results

### Parity-stratified analyses indicate different lead variants by parity

First, we explored for maternal or foetal genetic effect differences by parity on gestational duration and birth weight with parity-stratified GWASs using the Moba cohort (Supplementary Table 1).

**Figure 1:**
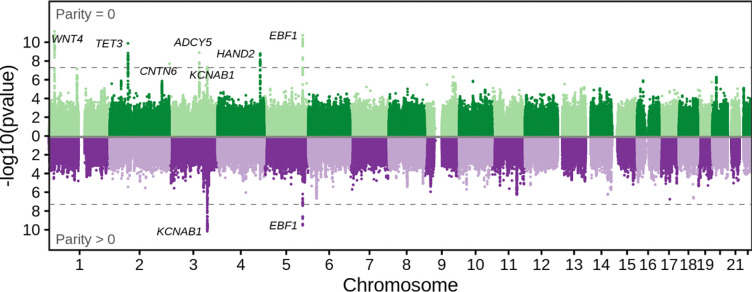
Miami plot illustrating the parity-stratified maternal GWASs on gestational duration. The top figure represents the GWAS on a mother’s first pregnancy (n= 24 284), while the bottom figure illustrates the GWAS for later pregnancies (n=31 202). The x-axis shows the chromosome position and y-axis the two-sided p-value of the GWAS. The dashed line represents the genome-wide significance threshold (p-value = 5 × 10^−8^). Variants reaching the genome-wide significance threshold have the nearest protein-coding gene displayed.

In the maternal GWAS of gestational duration seven loci reached genome-wide significance in the first pregnancy (parity = zero) (Figure 1, Supplementary Figure 1, Supplementary Table 2); six of these loci have been previously identified (*WNT4*, *EBF1*, *TET3*, *ADCY5*, *HAND2* and *KCNAB1)* (6). The lead variant at the newly discovered locus is intronic to *CNTN6*, but this gene has no obvious relationship to gestational duration*.* In later pregnancies (parity > zero), two loci reached the genome-wide significant threshold (located near *EBF1* and *KCNAB1*) (Figure 1); both were in high LD (r^2^ > 0.85) with the first-pregnancy variants in the same region. In the GWAS of the whole study population for the maternal genome on gestational duration, 11 loci reached genome-wide significance (Supplementary Figure 1, Supplementary Table 2). Four of these loci had never been associated with gestational duration before (6) (r^2^ < 0.1, variants near *NDST4*, *WWTR1*, *HMGA1* and *WT1*) and all other loci were either previously identified or identified in the parity-stratified GWASs. Only the locus at *CNTN6* was significant only in the first pregnancy GWAS and not in the whole study population GWAS. We highlight the lead variant at *WWTR1* (rs73155026, beta = −0.37 days, p-value = 1.6× 10^−8^, MAF = 36.6%), which is fine-mapped as a splice QTL and expression QTL for *WWTR1* in multiple tissues, supporting its role as the causal gene. Loss of *WWTR1* mRNA expression is observed in cytotrophoblasts from placentas of extremely preterm newborns (36).

For the foetal GWAS of gestational duration (Supplementary Figure 2, Supplementary Table 3), we identified one genome-wide significant locus in the first pregnancy (*ADCY5*) and none in the subsequent pregnancies. To note, the locus is intronic for *ADCY5*, which harbours maternal and foetal effects on both gestational duration and birth weight and foetal effects on placental weight, adding another layer of complexity in this region. A previous GWAS has shown that the locus associated with gestational duration colocalizes with *ADCY5* gene expression in the uterus and affects fat-mass-related traits in the maternal genome (6). For birth weight, the locus affects glucose-related traits in the foetal genome. The foetal GWAS of gestational duration in the whole sample identified three independent loci, one of which was previously reported (*IL1A/B*). The lead variant at the *LSM4* locus (rs4808779, beta = −0.39 days, p-value = 2× 10^−9^, MAF = 33.8%) is fine-mapped as an expression-QTL for *JUND* and *IQCN* genes in brain and immune cell types, respectively, with concordant effect direction (i.e., the allele associated with higher gestational duration is associated with higher gene expression). *JUND* is a transcription factor that has been suggested to play a role in the switch from quiescent to contractile phenotype in the human pregnant myometrium (37). This contrasts with the fact that the discovery has been performed using the foetal genome, highlighting the difficulties in teasing out causal genes in phenotypes shaped by maternal and foetal genomes.

For birth weight, the number of genome-wide significant loci was slightly larger in later pregnancies for both the maternal and foetal genomes (2 vs 5 and 11 vs 13, respectively, Supplementary Figure 3–4, Supplementary Table 4–5). All maternal loci (including those from the whole sample GWAS) had been previously identified (2), except for a signal at the *HLA-B* gene region associated both in later pregnancies and in the GWAS using the whole sample (Supplementary Figure 3 and Supplementary Table 4). With regards to the foetal GWAS of birth weight, one locus was newly discovered in the first pregnancy, which was also identified in the GWAS using the whole sample (Supplementary Figure 4 and Supplementary Table 5). The lead variant at this locus (rs8033275, beta = −0.05 z-score, p-value = 1.4 × 10^−10^, MAF = 22.5%) is in strong LD with two missense variants affecting *NEDD4*. This gene regulates IGF-1 signalling and controls mice growth during development (38). In the whole sample GWAS we identified a total of 33 loci, seven of which were newly discovered in this GWAS (*NEDD4*, *SLC2A1*, *SIK2*, *HLA-B*, *SLC35D1*, *MSI2*, and *EDEM3*) (2). As part of the identified novel loci, two lead SNPs were in strong LD with missense variants affecting *WDR78* (rs2755253, beta = 0.03 z-score, p-value = 9.2 × 10^−9^, MAF = 32.7%) and *EDEM3* (rs78444298, beta = −0.10 z-score, p-value = 4.7 × 10^−8^, MAF = 2.0%)*.* We also highlight the lead variant at the *MSI2* locus, which is fine-mapped as an expression QTL for the same gene in the placenta.

The correlation between the effects of the genome-wide significant variants from the parity-stratified GWASs were modest for gestational duration and birth weight (r = 0.81–0.88) (not assessed for foetal genetic effects on gestational duration due to a small number of significant loci). We did not observe variants with opposite effect direction when splitting by parity, suggesting, if anything, that parity affects the magnitude of effect (Supplementary Figure 5). However, we note that it is also possible that the variants have an effect in first pregnancies and a null effect in later pregnancies, or vice-versa (a distinction between difference of magnitude of effect and null effect cannot be made based solely on the correlation between effect sizes).

To ensure that the analyses were not influenced by factors related to fertility or selection bias, we conducted GWASs of participating with the first or later pregnancies (case-control study) for the maternal and foetal genome. No variants reached genome-wide significance (Supplementary Figure 6), indicating no gross genetic differences between first and later-born.

Overall, the parity-stratified GWASs highlighted shared and distinct loci for both gestational duration and birth weight, with no clear pattern between outcomes. However, genetic variants reaching genome-wide significance had the same effect direction in different pregnancies.

### Two genetic variants previously associated with gestational duration interact with parity

**Figure 2:**
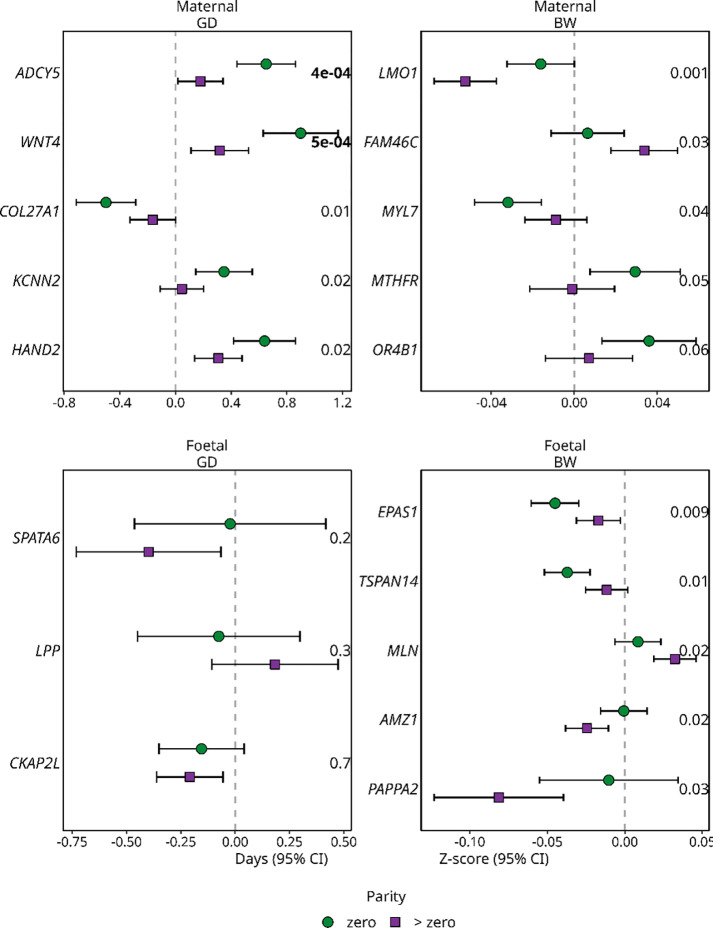
Parity-stratified effect sizes (n ~ 25,000 mothers/ children) for the variants with lowest interaction p-value (SNP × parity) for gestational duration (GD) and birth weight (BW) in the maternal and foetal genomes. The green circles are the effect sizes in first pregnancy and the purple square are the effect sizes in the later pregnancies. The error bars are the 95% confidence interval. Variants are displayed as the nearest protein-coding gene on the y-axis. The interaction p-value for each variant is displayed to the right. Only variants previously associated with the specific outcome in (2–6) were included (n_snps_ = 3 to n_snps_ = 189). For maternal gestational duration, maternal birth weight and foetal birth weight the remaining variants are illustrated in Supplementary Figure 7. Two genetic variants reached the Bonferroni-corrected significant threshold, with their p-values highlighted in bold (for p-value thresholds see Supplementary Table 7).

We then focused on the gene × parity interactions for genetic variants identified in previous studies (2–6) (n_snps_ = 3 to n_snps_ = 189) due to the comparatively sample size of our cohort and the multiple hypothesis-testing burden. Based on Bonferroni correction, adjusting for the total number of variants analysed for each phenotype and genome, two genetic variants for gestational duration reached the significance threshold for gene × parity interactions (Figure 2, Supplementary Figure 7, Supplementary Table 6 and p-value thresholds in Supplementary Table 7). Specifically, the top two variants for gestational duration in the maternal genome (*ADCY5* and *WNT4*) had a larger absolute effect size in the first pregnancy compared to later pregnancies.

*WNT4* is important for the development of the female reproductive system (39–41) and breast cancer (42). In mouse mammary cells, increasing parity decreased *WNT* signalling partly due to a more than threefold reduction in the *WNT4* ligand expression (43). This reduction of *WNT4* expression was believed to be linked to a decrease in the number of oestrogen/progesterone receptors in luminal cells in multiparous mice. Moreover, previous functional analysis of the *WNT4* locus associated with gestational duration indicated that the likely causative variant alters the binding of the *ESR1* oestrogen receptor by an improved binding capacity for the minor allele (44). If there is also a decrease in the number of oestrogen/progesterone receptors in uterus cells in multiparous women, it is possible that this variant plays a less significant role in later pregnancies compared to the first pregnancy.

### The cumulative effect of gestational duration-associated variants is modified by parity

Random forest models were developed to predict each of the two phenotypes using genetic variants previously associated with these traits (2–6). The aim was to investigate whether the aggregated genetic effects of lead variants differed by parity. We created random forest genetic scores to avoid splitting the data into training and testing sets, which would reduce the sample size available for testing the interaction between random forest genetic scores and parity.

**Figure 3:**
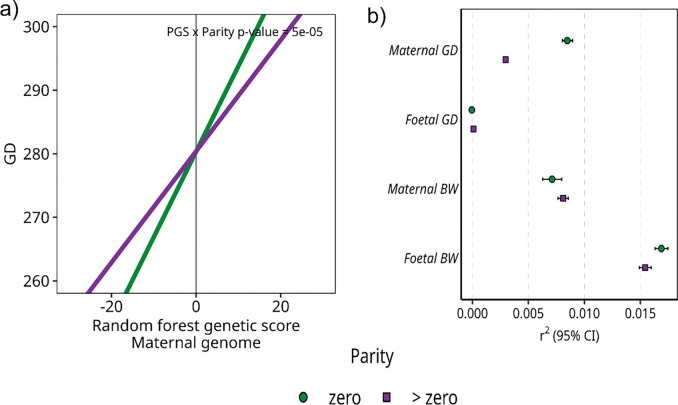
The interaction between the maternal random forest genetic score and parity on gestational duration, along with the prediction accuracy of each phenotype and genome in random forest models stratified by parity. a) The interaction between the maternal random forest genetic score and parity on gestational duration (GD). The slopes highlight the effect of the random forest genetic score on gestational duration by parity (green line: parity = 0 (n= 24 284), purple line: parity > 0 (n=31 202)) and are based on the estimates from the linear model used when testing the interaction (Supplementary Table 8). The x-axis shows the maternal random forest genetic score and the y-axis the gestational duration. The interaction p-value is displayed in the top right of the figure. b) Prediction accuracy (r^2^) of each phenotype and genome in random forest models stratified by parity (n ~ 25,000 mothers/ children) and constructed using hard-coded genotypes of variants reaching genome-wide significance in (2–6). Each point represents the prediction accuracy from one random forest model. The green circle is the prediction accuracy in first pregnancy and the purple square are the prediction accuracy in the later pregnancies. The 95 % credible interval was generated from 10 000 of r^2^ on out-of-bag predictions for the tuned hyperparameters in each model.

Among all the phenotype-genome combinations, only the random forest genetic scores of the maternal genome on gestational duration, derived from the whole population, showed a significant interaction with parity in a linear model (p-value interaction = 5 × 10^−5^, Supplementary Table 8). The interaction indicated that the effect of the random forest genetic score on gestational duration is larger in the first pregnancy compared to subsequent pregnancies (Figure 3a, Supplementary Table 8). Since the random forest genetic score only included genetic variants as features, this interaction indicates that known variants associated with gestational duration have a significantly higher genetic effect on gestational duration in the first pregnancy compared to later ones. Furthermore, in the random forest models derived from the whole population that included both genetic and environmental features the interaction for gestational duration in the maternal genome remained significant (Supplementary Table 9).

When splitting by parity, the random forest genetic scores had a higher predictive accuracy (r^2^) for the maternal genome on gestational duration in the first pregnancy and foetal genome on birth weight compared to subsequent ones (the 95% credible intervals did not overlap) (Figure 3b, Supplementary Table 10). Conversely, for gestational duration in the foetal genome and birth weight on the maternal genome, we observed higher predictive accuracy in later pregnancies. Notably, the largest fold change in prediction accuracy between first and subsequent pregnancies was observed for gestational duration in the maternal genome (Supplementary Table 10). Overall, the predictive accuracy of the random forest genetic scores was low for all outcomes, except for the foetal genome on birth weight.

Across all phenotypes and all genomes, the prediction accuracy was higher when including both genetic and environmental features compared to including only genetic variants, except for gestational duration on the foetal genome in first pregnancies where there was no difference between the two feature sets (Supplementary Table 10). The trait with the lowest improvement in prediction accuracy when including both genetic and environmental features was gestational duration in the maternal genome (Supplementary Table 10). This may indicate that gestational duration has an unresolved complexity compared to birth weight. In the parity-stratified study population, the predictive accuracy of the maternal genome on gestational duration was also higher in the first pregnancy, following the same pattern as when using only genetic features (Supplementary Table 10).

Overall, the random forest models indicated diverse patterns for each combination of genome-phenotype. The largest difference in the aggregated genetic effect of previously known variants by parity was observed for the maternal genome on gestational duration, with larger genetic effects in first pregnancies compared to later ones.

### Parity-dependent effect sizes and differences in the total genetic variance by parity

**Figure 4:**
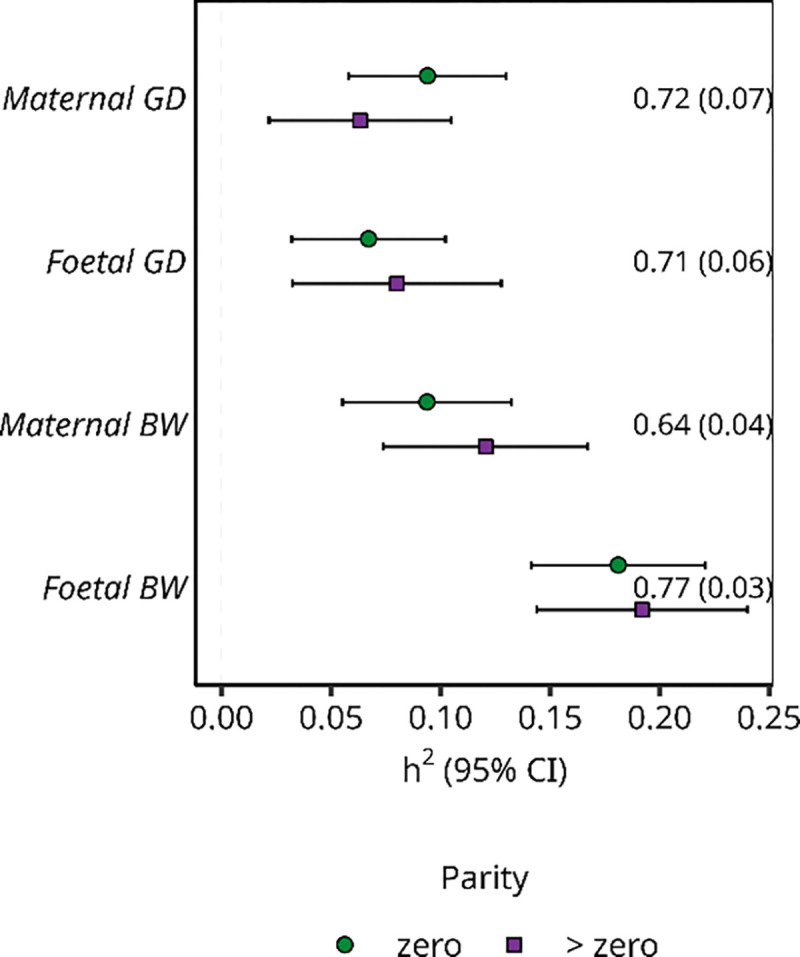
SNP heritability for gestational duration (GD) and birth weight (BW) in the maternal and foetal genomes by parity (n ~ 25,000 mothers/ children). The green circles are the heritability estimates in first pregnancy and the purple square are the heritability estimates in the later pregnancies. The error bars are the 95% confidence interval. On the right side of the figure, the genetic correlation between parity = zero and parity > zero is displayed with its standard error within the parentheses.

Next, we estimated genetic correlations and heritability for each phenotype and between parities using LD score regression (28, 29). The genetic correlation between the GWAS in first pregnancies and GWAS in later pregnancies for any phenotype using either the maternal or foetal genome ranged from 0.64 – 0.77 (Figure 4). A genetic correlation deviating from one indicates parity-dependent effect sizes that are not proportional and/or parity-dependent loci. Compared to the correlations reported in (45), which analysed 33 traits and 10 environmental variables for gene × environment interactions (with genetic correlations ranging from 0.85 to 0.98), the correlations in this study are relatively low implying parity-specific genetic effects. Furthermore, the imperfect genetic correlations are in concordance with the parity-stratified GWASs, which suggested different lead SNPs by parity (Figure 1, Supplementary Figures 1–4).

There were no differences by parity in the parity-stratified SNP heritability estimates for any of the two phenotypes using the maternal or foetal genomes (Figure 4). However, the phenotypic variance for gestational duration was largest in first pregnancies and that for birth weight larger in subsequent pregnancies (Supplementary Table 1); the largest absolute difference was again observed for gestational duration. Considering the narrow sense heritability equation $h^{2}=\frac{V_{A}}{V_{P}}$; since the phenotypes show differences in the phenotypic variance ($V_{P}$) by parity (Supplementary Table 1) but no difference in heritability ($h^{2}$), this indicates differences in the genetic variance ($V_{A}$) in both gestational duration and birth weight. Given that we observed no differences in heritability by parity for any outcome, we speculate that the genetic variance must be larger for gestational duration in the first pregnancy due to the larger phenotypic variance. Conversely, for birth weight, later pregnancies have a larger genetic variance. This result is in line with the number of discovered loci for both gestational duration and birth weight.

## Discussion

In previous GWASs of gestational duration and birth weight the number of previous deliveries was not considered. In this study, we investigated whether parity modifies the maternal or foetal genetic effects on these pregnancy outcomes. Our findings show that the identified loci and their numbers were moderately different when stratifying the GWASs by parity, with stronger maternal genetic effects on gestational duration. Moreover, the genetic correlations between first and later pregnancies deviated from one for both gestational duration and birth weight.

We observed the strongest indication of effect modification by parity for the maternal genome on gestational duration. Our findings suggest that the magnitude of genetic effects is larger in the first pregnancy, particularly for variants with an already known association with gestational duration. However, the molecular mechanisms regulating the timing of delivery in humans are poorly understood, complicating the biological interpretation of these results. During a woman’s first pregnancy and delivery, numerous physiological first-time changes occur in the body, this could possibly explain the larger genetic effects for gestational duration in the maternal genome. One example is the growth and expansion of the uterus. Parity is associated with smaller uterine size in the first pregnancy (12) (46). After the first pregnancy, the muscle fibres become stretched and will more easily expand for further pregnancies, which can result in the genetic control to expand the uterus being less important in later pregnancies. This is supported by the variant near *WNT4*, which in our study showed a larger effect in the first pregnancy and has been correlated with an increase in uterine size by 35% in mice (47). It is difficult to conclude anything about the foetal effects on gestational duration given the few loci known to be associated with this phenotype.

While our findings for gestational duration were robust, the picture for birth weight is blurrier. Birth weight variance is larger in offspring of later pregnancies; it is therefore reasonable to assume that the genetic effects on birth weight are also stronger in later pregnancies, because we found no differences in heritability estimates. In concordance with this, we have identified larger number of loci in the GWAS of birth weight in both the maternal and foetal genomes. However, we observe the opposite in previously identified loci in the foetus, genome which contributes more to the heritability of birth weight compared to the maternal genome (48). However, this may have arisen as a bias induced by the fact that data from previous GWAS on birth weight were largely based on data from the UK Biobank, where birth weight data is from the first pregnancy. Moreover, we know that there is an interplay between the two studied phenotypes, where gestational duration is a key determinant of birth weight. As previously discussed, we hypothesise that the genetic control to expand the uterus does not need to be as large in later pregnancies since the uterus expands more easily after the first pregnancy. Animal studies have shown that uterine capacity influences birth weight; for instance, large genotype lambs were lighter and smaller when born to small genotype dams (49). Easier expansion of the uterus in later pregnancies may also result in greater flexibility for the foetus to adjust its growth. This could be one reason for larger mean birthweight in later pregnancies, and maybe partly explain the larger genetic effect in later pregnancies for birth weight.

Our results highlight the importance of considering parity in genetic studies of pregnancy outcomes. We suggest that parity modifies the genetic variance of gestational duration and birth weight. It is important to study a homogeneous population, and parity should be considered in future genetic research related to pregnancy outcomes. However, the difference in genetic effects by parity on all phenotype-genotype combination needs to be replicated in other cohorts from both European and non-European ancestries to strengthen the results shown in this paper. A strength of this study is the availability of genotype data from both mothers and offspring in >50,000 samples in a single cohort; however, the sample size is comparatively small to genetic studies of non-pregnancy phenotypes. Increasing sample size might reveal additional loci with different effects across pregnancies and might help resolve parity effects that in this study were difficult to interpret, particularly in relation to birth weight.

To conclude, we have provided evidence that parity modifies the genetic effect on gestational duration. The strongest evidence of modification effect was found for gestational duration in the maternal genome where genetic effects overall are stronger in the first pregnancy compared to later ones.

## Supplementary Material

Supplement 1

Supplement 2

## Figures and Tables

**Table 1 –:** Sample sizes of the 12 genome-wide association studies preformed

Phenotype	Genome	n _whole study population_	n _parity = zero_	n _parity > zero_
Gestational duration	Maternal	55 486	24 284	31 202
	Foetal	50 728	22 184	28 544
Birth weight	Maternal	49 052	21 353	27 699
	Foetal	58 528	25 412	33 116

**Table 2 –:** Description of random forest models (per phenotype-genome combination)

Model description		
Population	Features	Outcome
Whole study population	Genetics only	Residualised
Parity = zero	Genetics only	Residualised
Parity > zero	Genetics only	Residualised
Whole study population	Genetics and environmental	Non-residualised
Parity = zero	Genetics and environmental	Non-residualised
Parity > zero	Genetics and environmental	Non-residualised

**Table 3 -:** Genetic variants previously associated with the phenotypes in this study’s genotype data

Phenotype	Genome	Top SNPs still missing	Number of Top SNPs left
Gestational duration	Maternal	5*	27
	Foetal	1	3
Birth weight	Maternal	4	74
	Foetal	10**	189

* Note that for the maternal genome for gestational duration two of the missing SNPs are located on the X chromosome, which is not included in the MoBaPsychGen genotypes.

** Note that for the foetal genome for birth weight three of the missing SNPs are located on the X chromosome, which is not included in the MoBaPsychGen genotypes.
