# Supplementary material for: Parity modifies the effect of genetic variants associated with gestational duration and birth weight
Source: medRxiv. 2025 Jun 17:2025.06.17.25329777. Preprint. [Version 1] doi: 10.1101/2025.06.17.25329777 (PMC12204269; doi:10.1101/2025.06.17.25329777)
Supplement: Supplement 2 [file media-2.pdf]

Supplementary Method

To optimize the performance of the random forest the following hyperparameters were tuned: number of decision trees (n trees), the ratio of features that were available to be considered at each split (mtry ratio), minimum number of observations in a leaf node (min node size) and mechanism used for node splitting (splitrule). In addition, subsampling without replacement (replace) of different fractions (sample fraction) was also tested instead of replacement (bootstrap sampling), since the out-of-bag error in random forests can overestimate the actual test error (1, 2). The search space used for hyperparameters were:

- n trees: Any integer between 20 and 1000
- mtry ratio: Any float between 0 and 1
- min node size: Any integer between 1 and 40
- splitrule: "variance", "extratrees" and "maxstat"
- replace: True or False
- Sample fraction: Any float between 0.1 and 1

Tuning was done using Bayesian optimization. The set of hyperparameters with the highest r-squared out-of-bag-score was selected for each model (Table M1).

The same set of data is used both for tuning hyperparameters and evaluating model performance. The out-of-bag samples are selected randomly which means that it is possible that the samples used for testing and selecting hyperparameters are used again for testing when evaluating the model performance, thus causing an overestimation of the model performance due to overfitted hyperparameters. While running the evaluation multiple times reduces the risk of this having any effect on the overall results, we wanted to be certain that this is the case. We created a new setup using nested cross-validation. This method is more time consuming and computationally heavier, but it ensures that the test set used for evaluating the model has never been seen by the model. We used this method on a subset of the different models (gestational duration maternal genome for both feature sets) and compared the results to those obtained with our original setup. As seen in the Table M2 the difference in model performance is very small, suggesting that the risk of overestimating model performance is very small.

Table M1 – Optimal hyperparameters for the random forest models

Model description				Hyperparameters					
Features[a]	Phenotype[b]	Genome	Parity	n trees	mtry ratio	min node size	splitrule	replace	Sample fraction
G	gd	Maternal	Whole population	669	0.253	40	extratrees	TRUE	0.444
G	bw	Maternal	Whole population	714	0.631	40	extratrees	TRUE	0.326
G	gd	Foetal	Whole population	409	0.377	38	maxstat	TRUE	0.455
G	bw	Foetal	Whole population	946	0.907	24	variance	FALSE	0.205
G	gd	Maternal	0	669	0.253	40	extratrees	TRUE	0.444
G	bw	Maternal	0	903	0.888	32	extratrees	TRUE	0.170
G	gd	Foetal	0	61	0.583	2	maxstat	TRUE	0.204
G	bw	Foetal	0	914	0.878	6	extratrees	TRUE	0.396
G	gd	Maternal	> 0	825	0.902	11	maxstat	TRUE	0.479
G	bw	Maternal	> 0	803	0.982	38	extratrees	TRUE	0.327
G	gd	Foetal	> 0	577	0.597	37	maxstat	TRUE	0.978
G	bw	Foetal	> 0	926	0.907	10	variance	TRUE	0.177
GE	gd	Maternal	Whole population	669	0.253	40	extratrees	TRUE	0.444
GE	bw	Maternal	Whole population	590	0.964	19	extratrees	TRUE	0.101
GE	gd	Foetal	Whole population	881	0.907	40	maxstat	TRUE	0.360
GE	bw	Foetal	Whole population	415	0.667	30	extratrees	FALSE	0.446
GE	gd	Maternal	0	815	0.210	40	extratrees	TRUE	0.369
GE	bw	Maternal	0	546	0.878	25	extratrees	TRUE	0.106
GE	gd	Foetal	0	29	0.721	34	maxstat	FALSE	0.950
GE	bw	Foetal	0	815	0.579	40	extratrees	FALSE	0.455
GE	gd	Maternal	> 0	669	0.253	40	extratrees	TRUE	0.444
GE	bw	Maternal	> 0	425	0.669	31	extratrees	TRUE	0.200
GE	gd	Foetal	> 0	478	0.911	24	maxstat	TRUE	0.318
GE	bw	Foetal	> 0	331	0.847	27	extratrees	FALSE	0.303

[a] G = genetic features only, GE = genetic and environmental features

[b] gd = gestational duration, bw = birth weight

Table M2 – Model performance (r^2) using Bayesian optimization and nested cross-validation respectively

Model description				r^2 (95% credible interval)	
**Features[a]**	**Phenotype[b]**	**Matern al / Foetal**	**parity**	**Bayesian optimization**	**Nested cross-validation**
G	gd	Matern al	Whole population	0.00602 (0.00571 - 0.00632)	0.00916 (0.00881 - 0.00975)
G	gd	Matern al	0	0.00848 (0.00802 - 0.00895)	0.0121 (0.0118 - 0.0137)
G	gd	Matern al	> 0	0.00305 (0.00294 - 0.00315)	0.00671 (0.00577- 0.00754)
GE	gd	Matern al	Whole population	0.00943 (0.00908 – 0.00978)	0.0120 (0.0113 – 0.0123)
GE	gd	Matern al	0	0.0108 (0.0103 – 0.0113)	0.0139 (0.0126 – 0.0148)
GE	gd	Matern al	> 0	0.00777 (0.00732 – 0.00824)	0.00951 (0.00872 – 0.00997)

[a] G = genetic features only, GE = genetic and environmental features

[b] gd = gestational duration, bw = birth weight

References Supplementary Method

1. Janitza S, Hornung R. On the overestimation of random forest's out-of-bag error. PLOS ONE. 2018;13(8):e0201904.
2. Mitchell M. Bias of the Random Forest Out-of-Bag (OOB) Error for Certain Input Parameters. Open Journal of Statistics. 2011;01:205-11.

Supplementary Figures

a) First pregnancy (parity = zero) maternal genome gestational duration

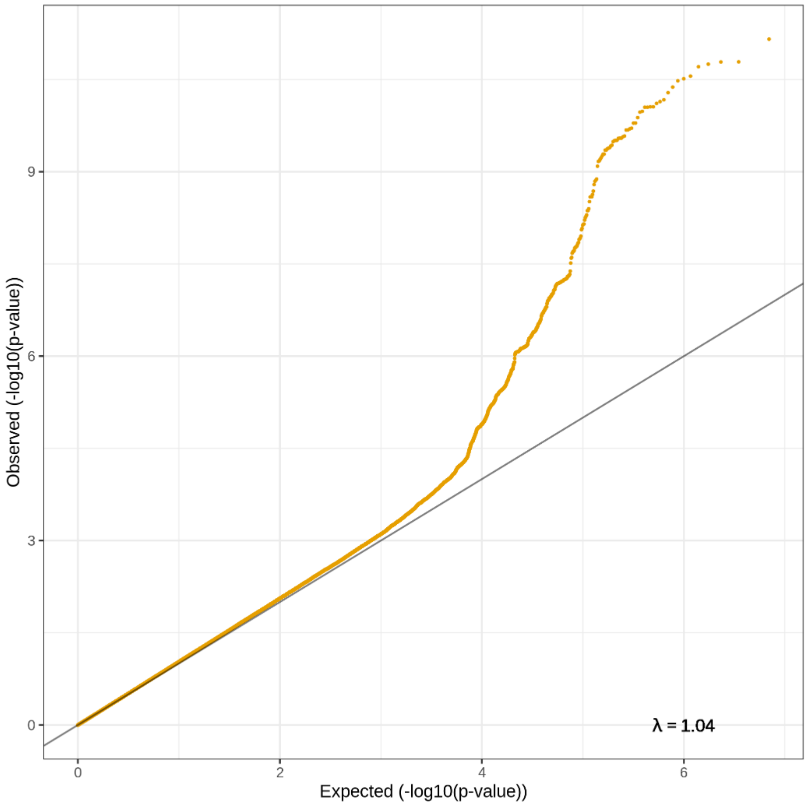

b) Later pregnancies (parity > zero) maternal genome gestational duration

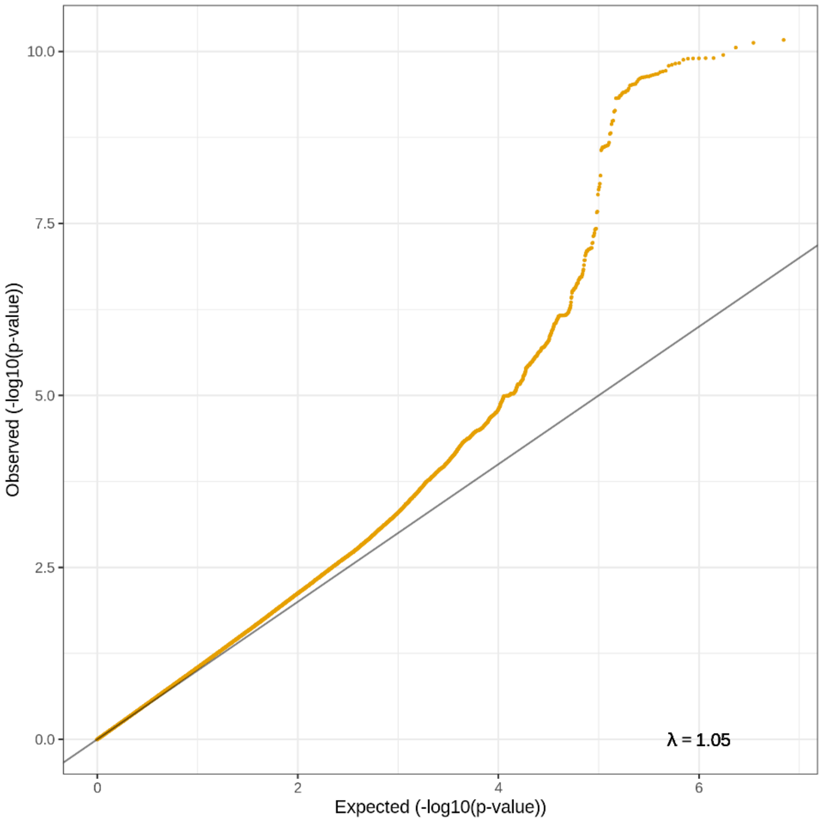

c) Whole study population maternal genome gestational duration

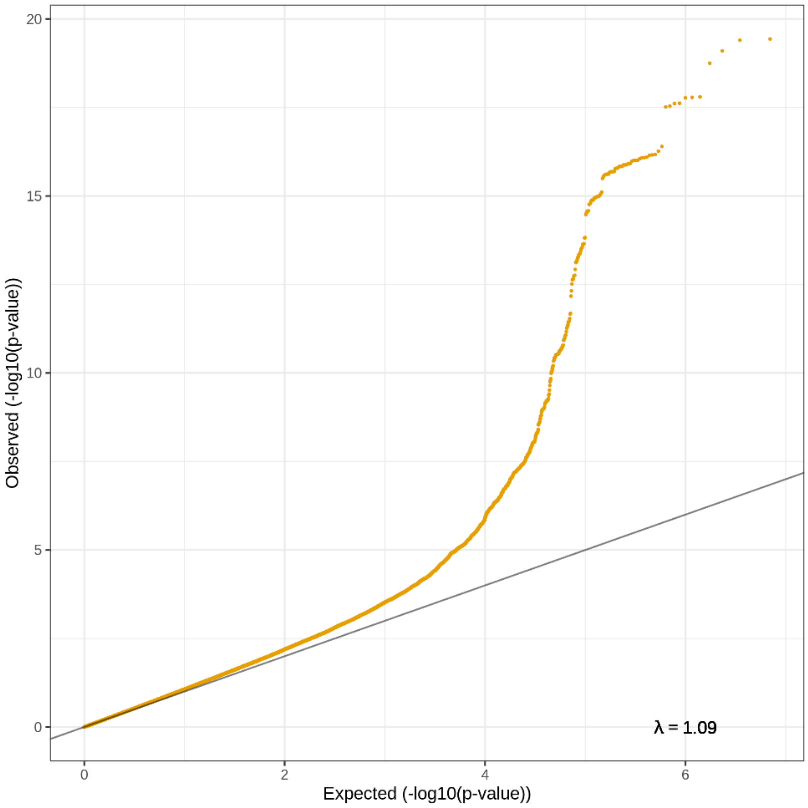

d) Whole study population maternal genome gestational duration

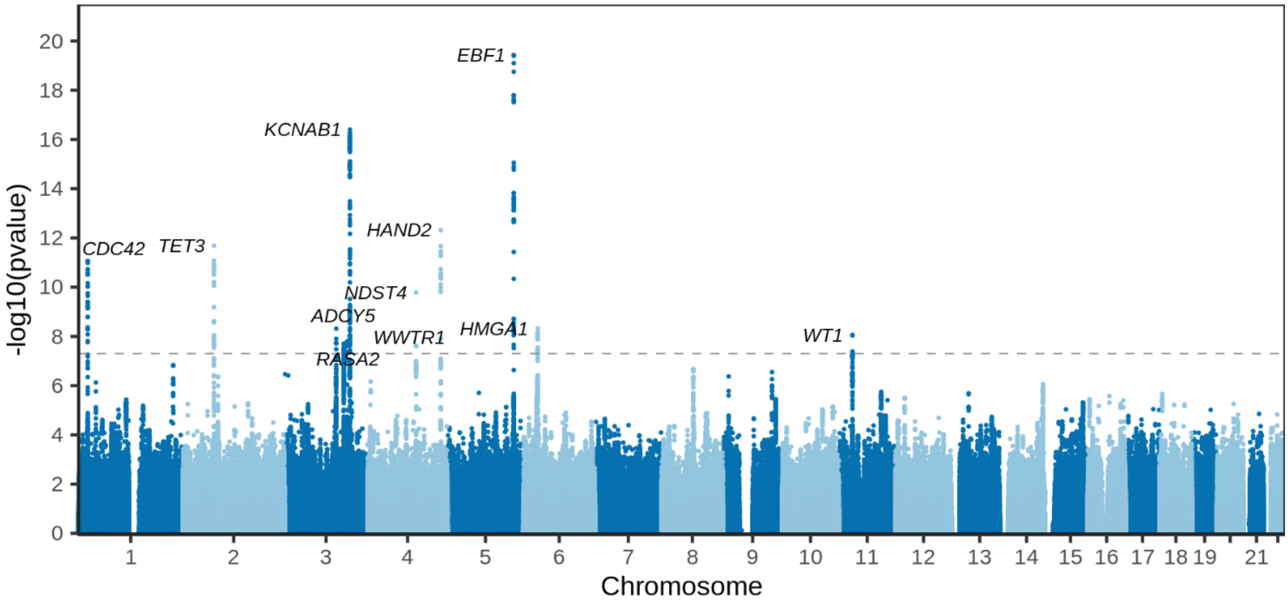

Supplementary Figure 1. QQ-plots and a Manhattan plot of the gestational duration genome-wide association studies on the maternal genome. The QQ-plots shown for a) parity = zero, b) parity > zero, and c) the whole study population include the lambda genomic control and demonstrate little to no test statistic inflation. In d), the Manhattan plot for the whole study population, the dashed line represents the genome-wide significant level (p-value = 5 x 10^{-8}). The x-axis shows the chromosome position and y-axis the two-sided p value of the GWAS. Variants reaching the significance threshold have the nearest protein-coding gene displayed.

a) First pregnancy (parity = zero) foetal genome gestational duration

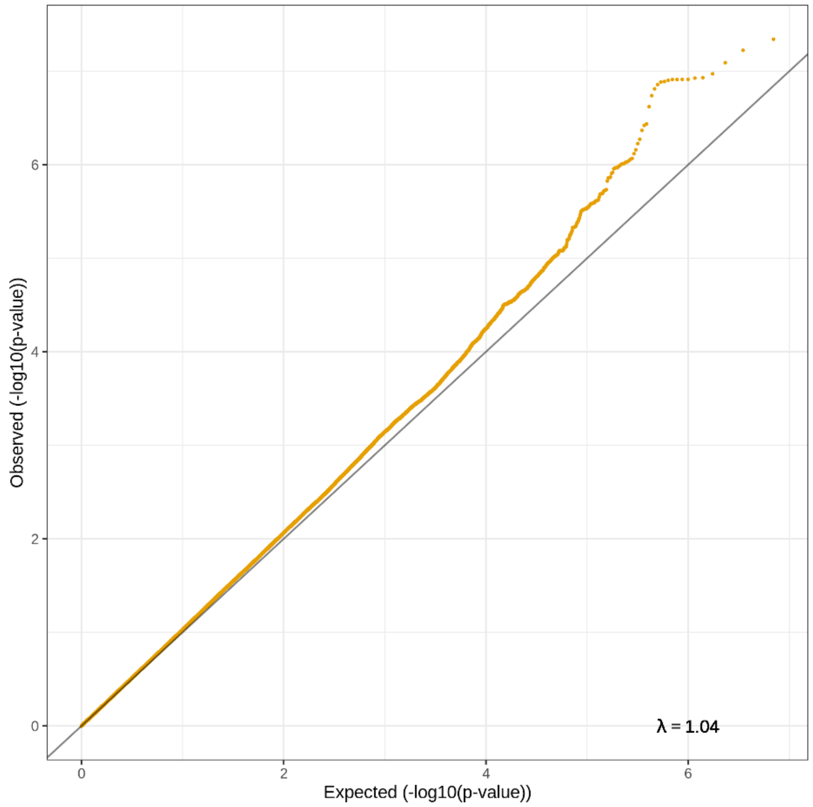

b) Later pregnancies (parity > zero) foetal genome gestational duration

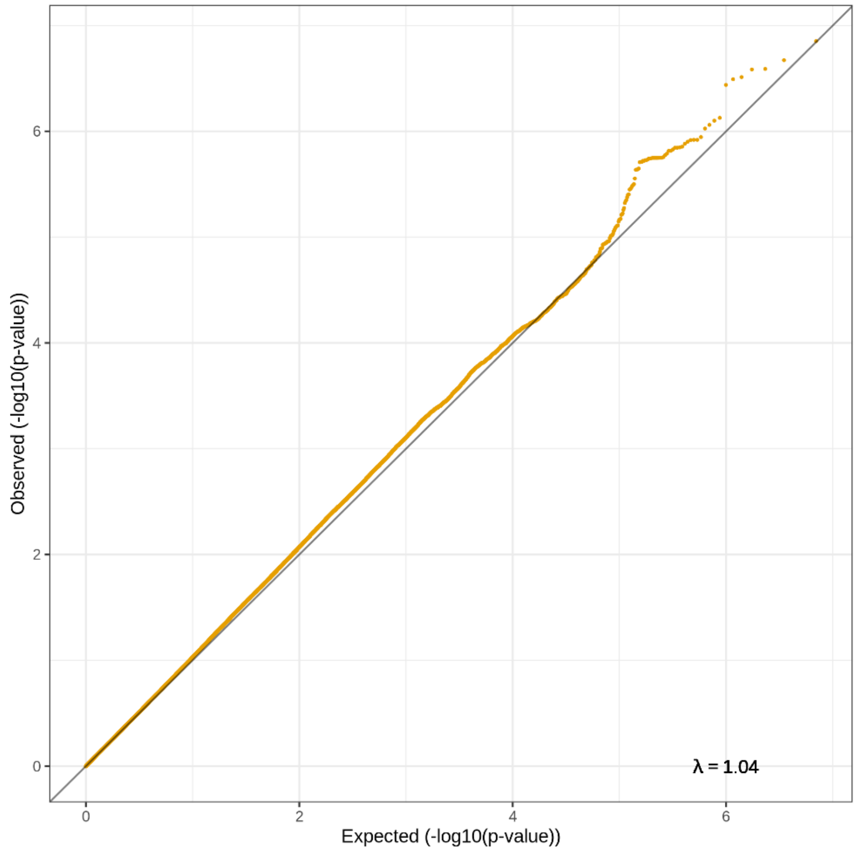

c) Whole population foetal genome gestational duration

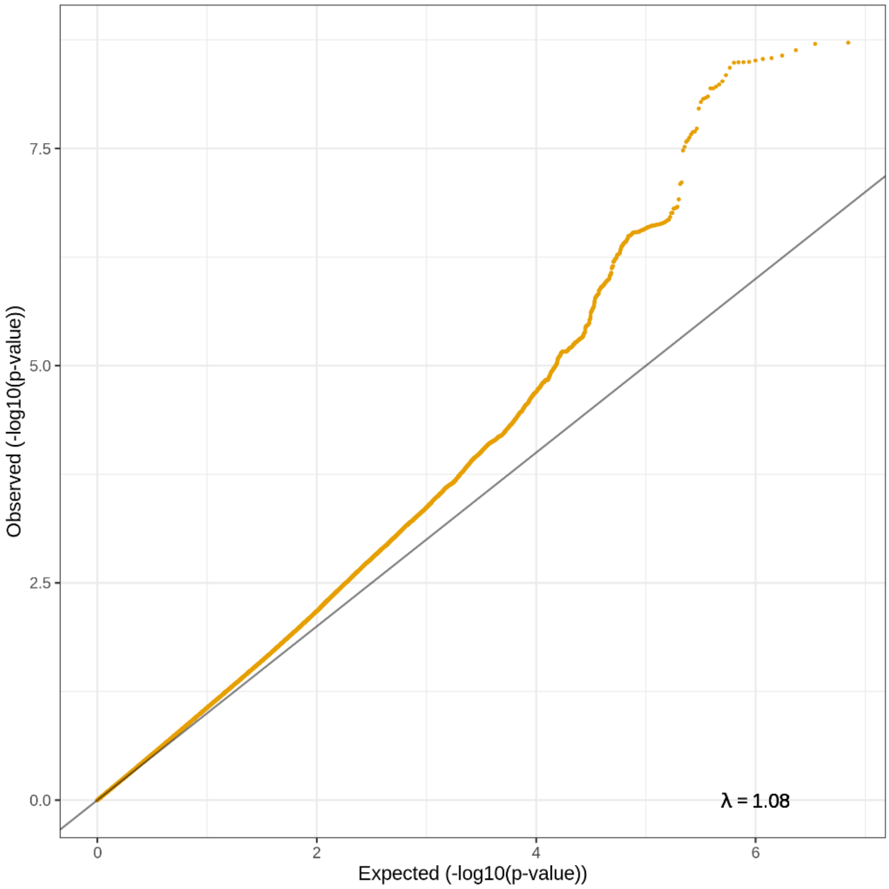

d) First pregnancy (top) and later pregnancies (bottom), foetal genome gestational duration

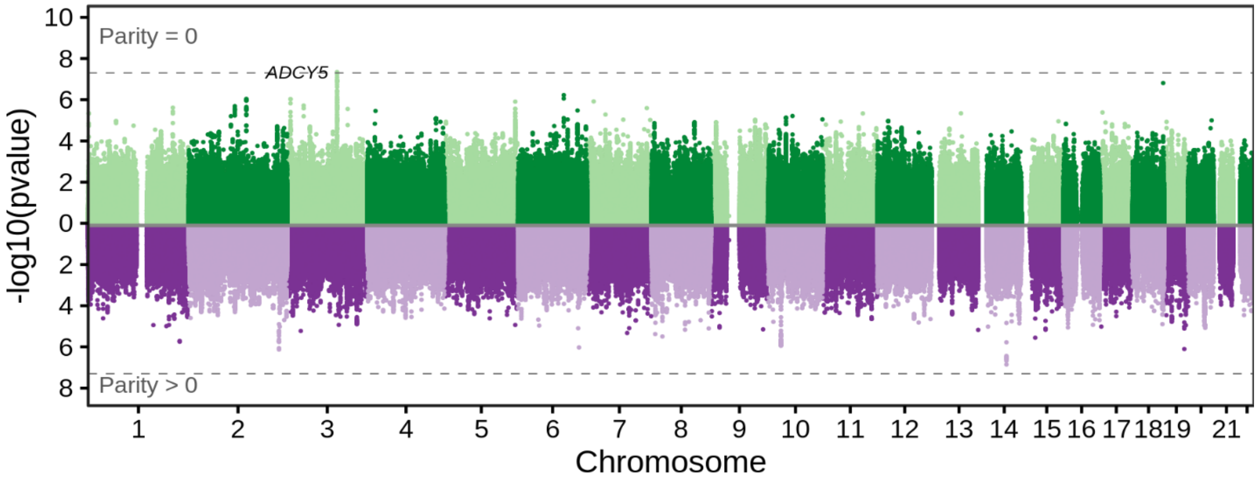

e) Whole study population foetal genome gestational duration

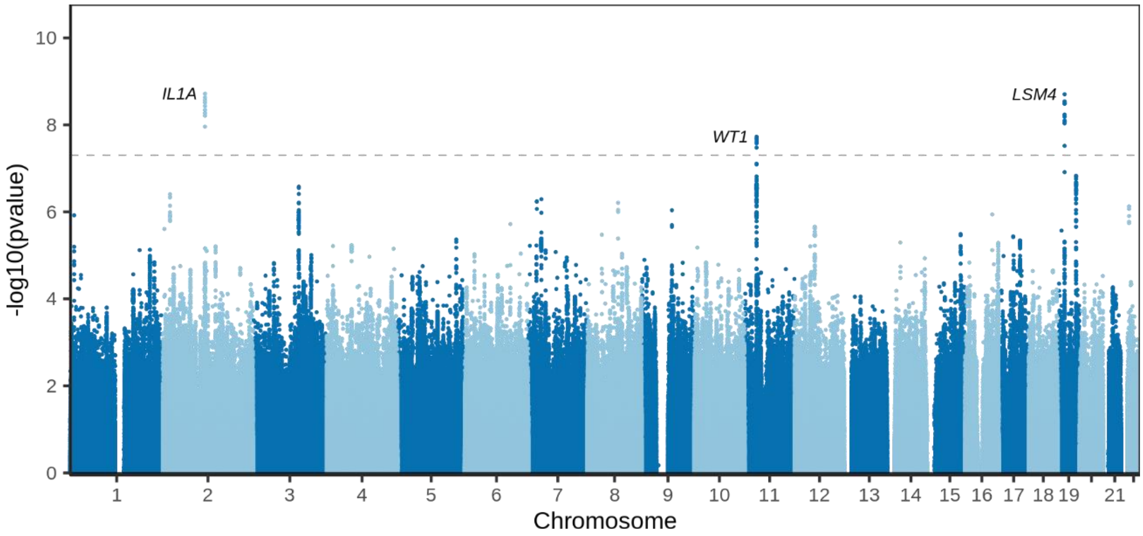

Supplementary Figure 2. QQ-plots and Manhattan plots of gestational duration genome-wide association studies on the foetal genome. The QQ-plots shown for a) parity = zero, b) parity > zero, and c) the whole study population include the lambda genomic control and demonstrate little to no test statistic inflation. In the Miami plot for d) first pregnancy (parity = zero, top, green) and later pregnancies (parity > zero, bottom, purple), and the Manhattan plot for e) the whole study population, the dashed line represents the genome-wide significant level (p-value = 5 x 10^{-8}). The x-axis shows the chromosome position and y-axis the two-sided p value of the GWAS. Variants reaching the significance threshold have the nearest protein-coding gene displayed.

a) First pregnancy (parity = zero) maternal genome birth weight (z-score)

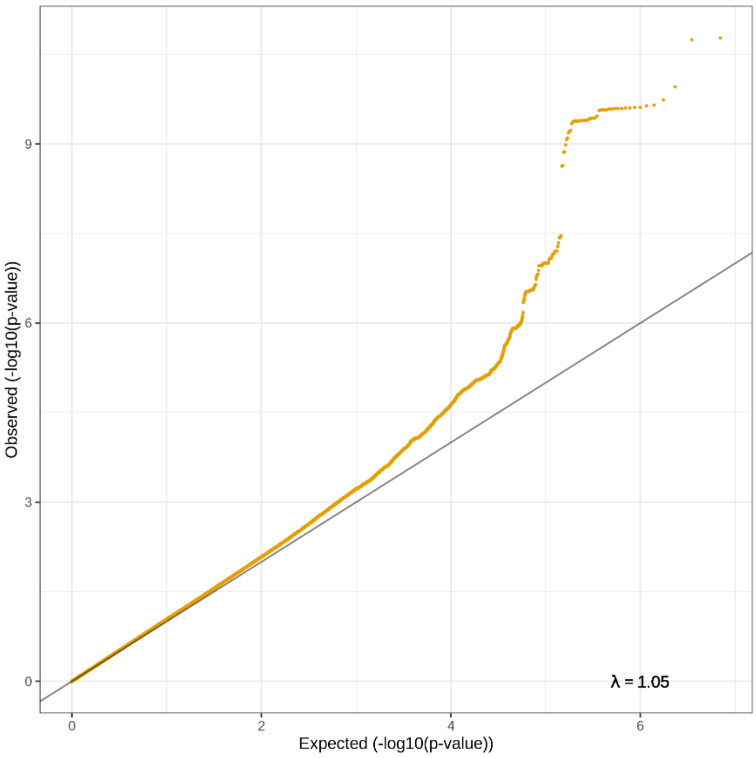

b) Later pregnancies (parity > zero) maternal genome birth weight (z-score)

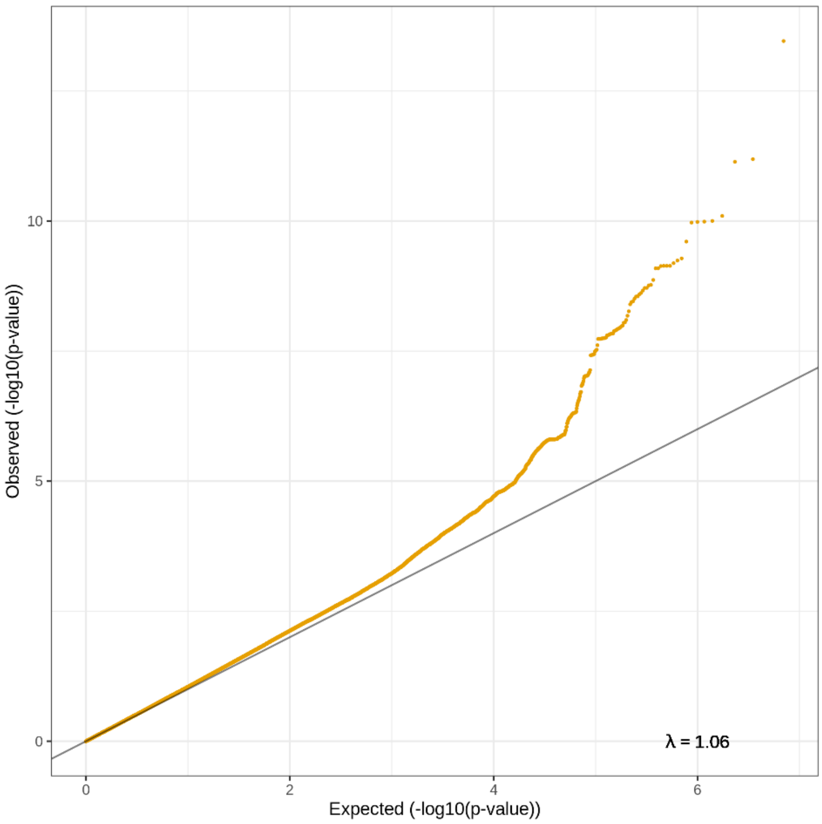

c) Whole population maternal genome birth weight (z-score)

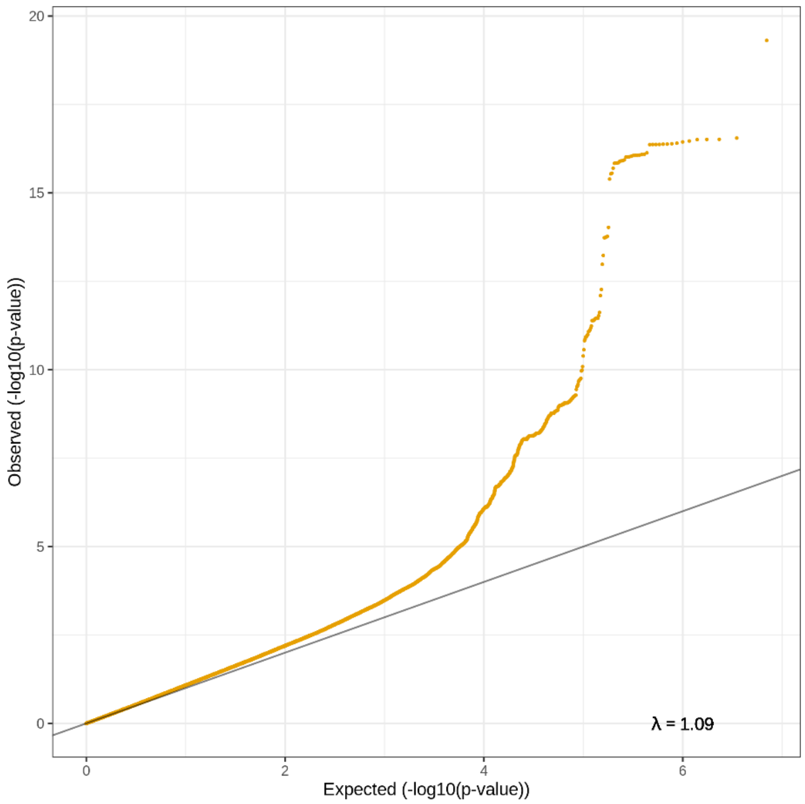

d) First pregnancy (top) and later pregnancies (bottom), maternal genome birth weight (z-score)

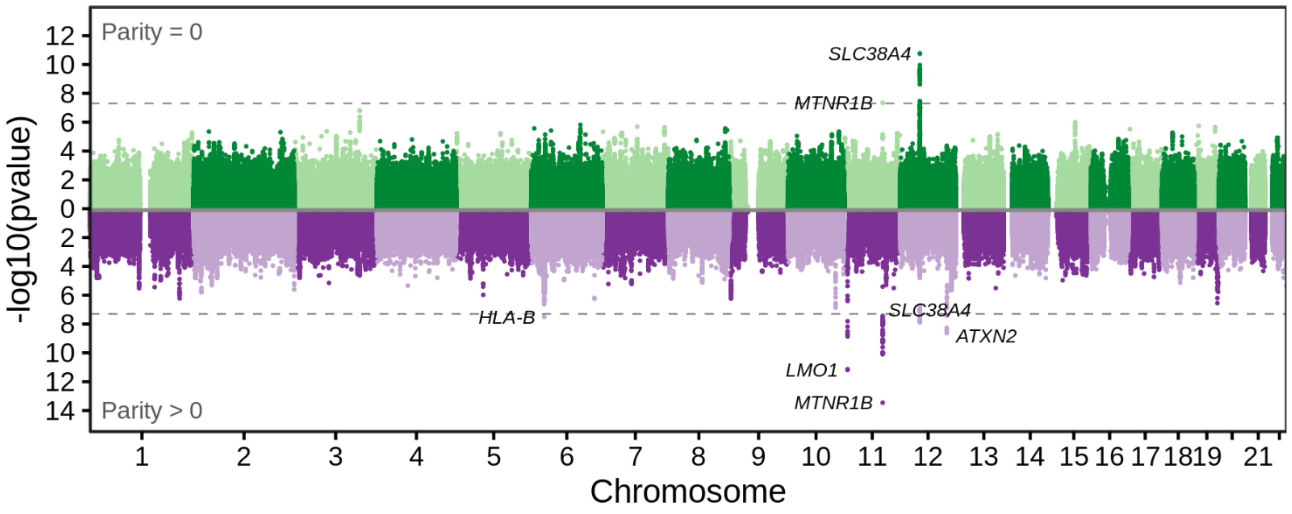

e) Whole study population maternal genome birth weight (z-score)

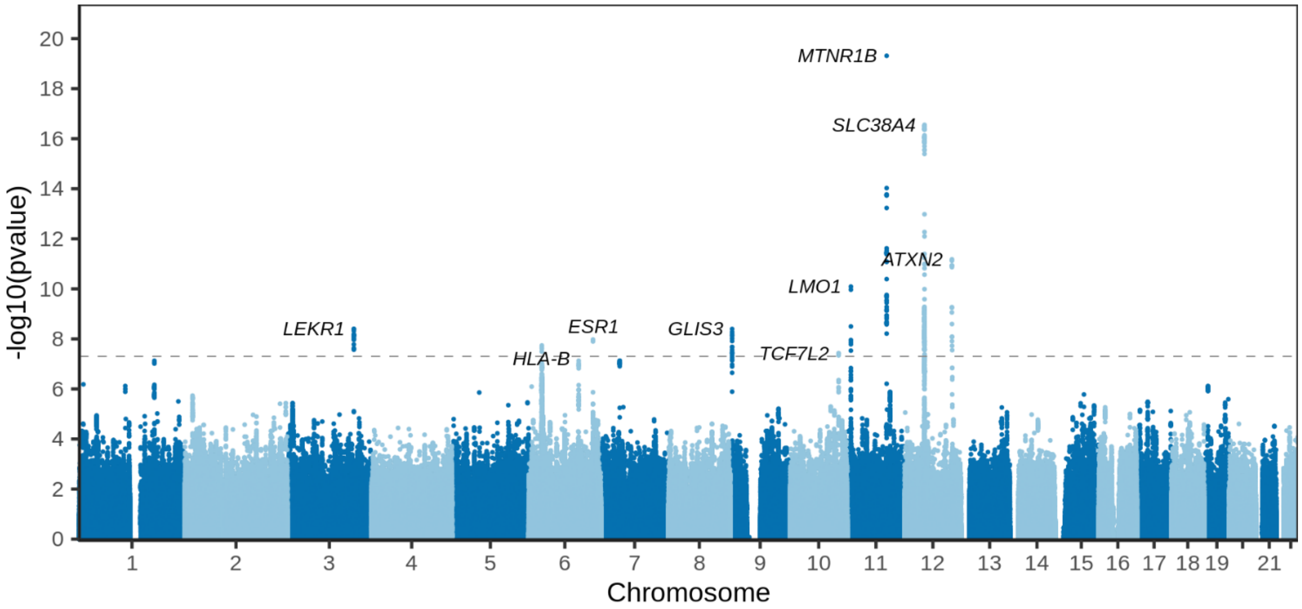

Supplementary Figure 3. QQ-plots and Manhattan plots of birth weight (z-score) genome-wide association studies on the maternal genome. The QQ-plots shown for a) parity = zero, b) parity > zero, and c) the whole study population include the lambda genomic control and demonstrate little to no test statistic inflation. In the Miami plot for d) first pregnancy (parity = zero, top, green) and later pregnancies (parity > zero, bottom, purple), and the Manhattan plot for e) the whole study population, the dashed line represents the genome-wide significant level (p-value = 5 x 10^{-8}). The x-axis shows the chromosome position and y-axis the two-sided p value of the GWAS. Variants reaching the significance threshold have the nearest protein-coding gene displayed.

a) First pregnancy (parity = zero) foetal genome birth weight (z-score)

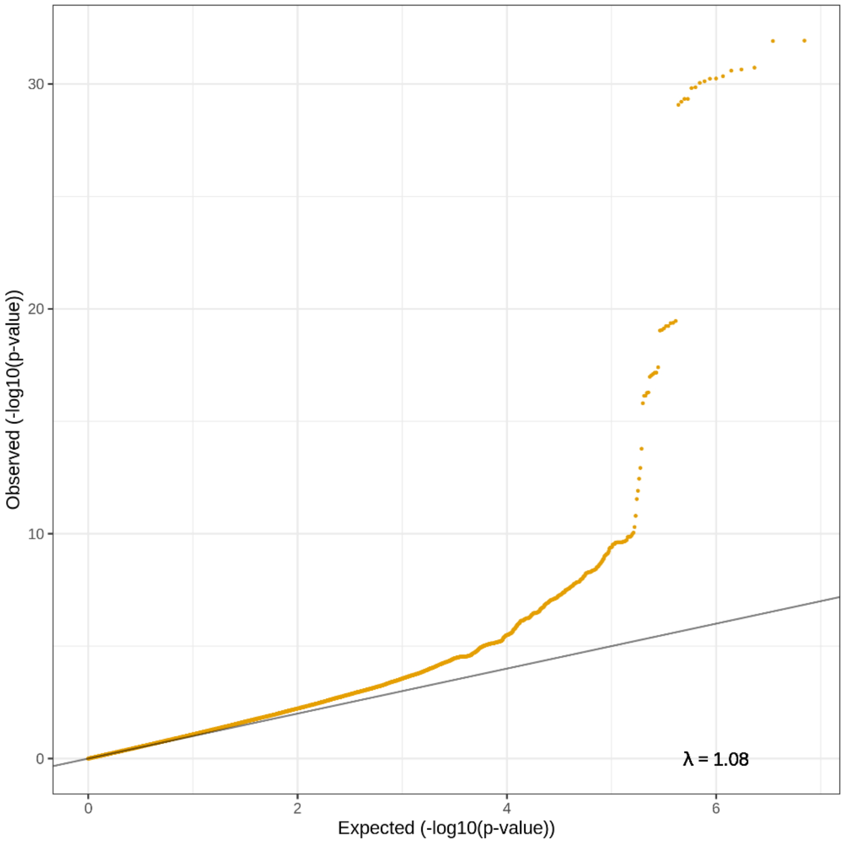

b) Later pregnancies (Parity > zero) foetal genome birth weight (z-score)

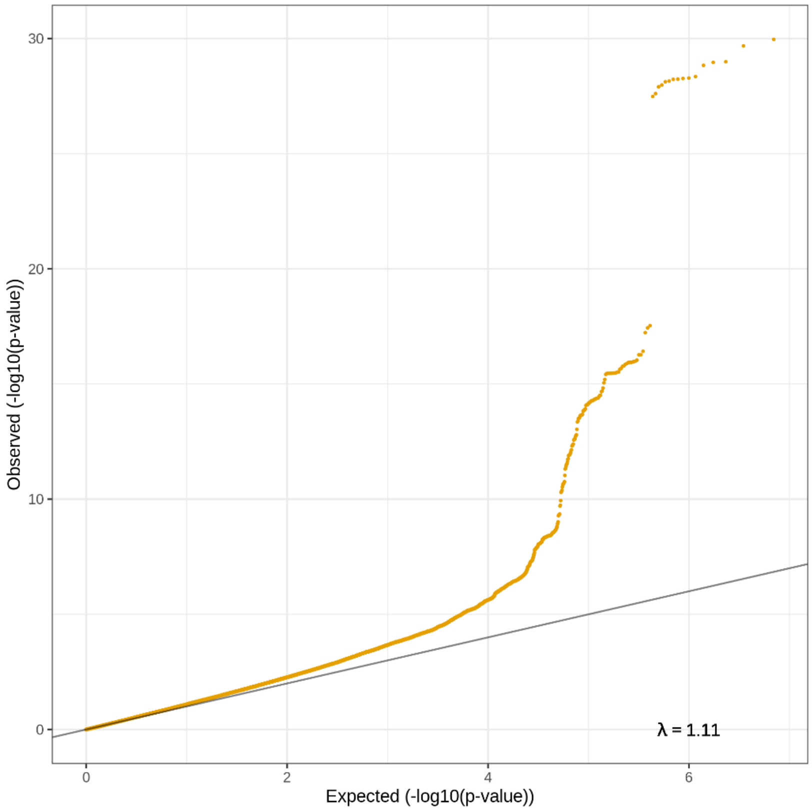

c) Whole population foetal genome birth weight (z-score)

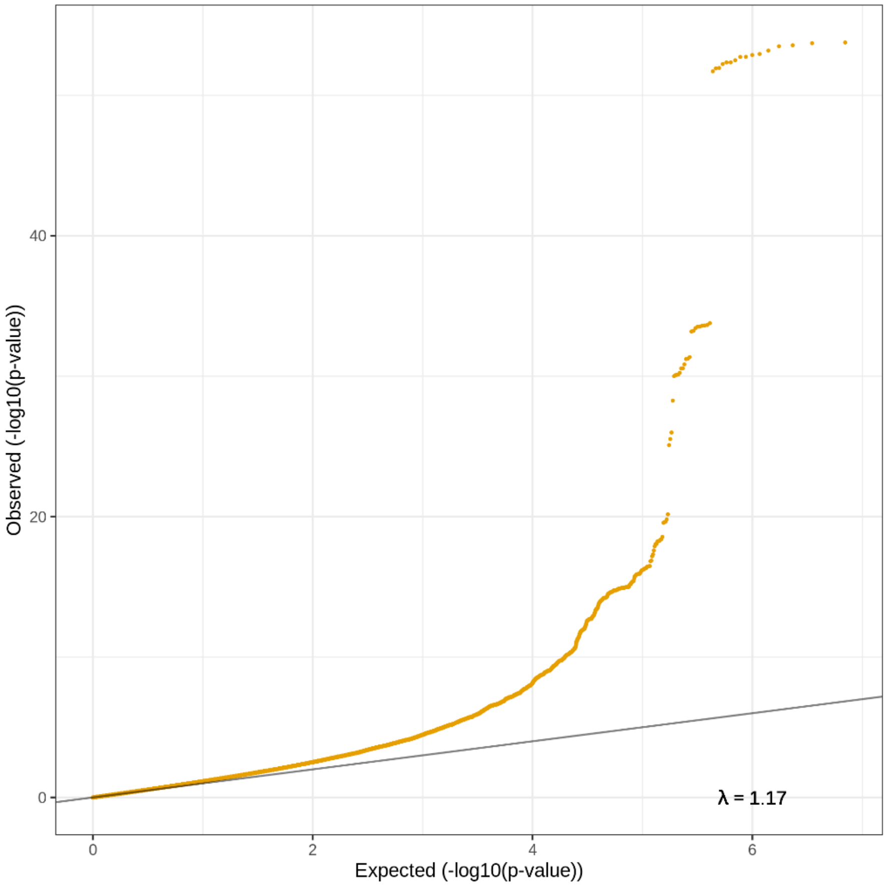

d) First pregnancy (top) and later pregnancies (bottom), foetal genome birth weight (z-score)

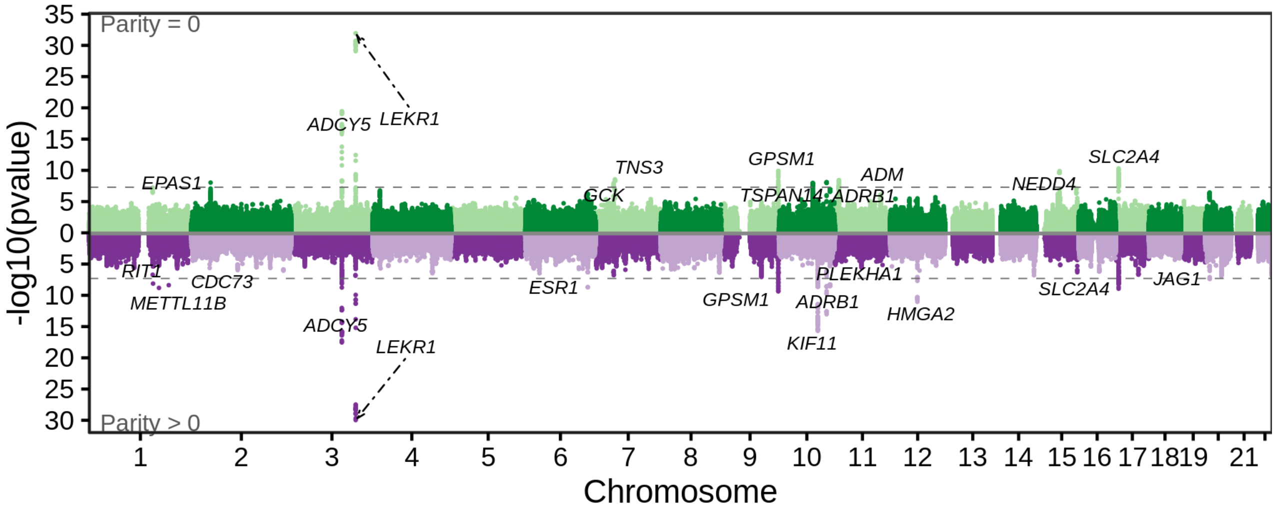

e) Whole study population foetal genome birth weight (z-score)

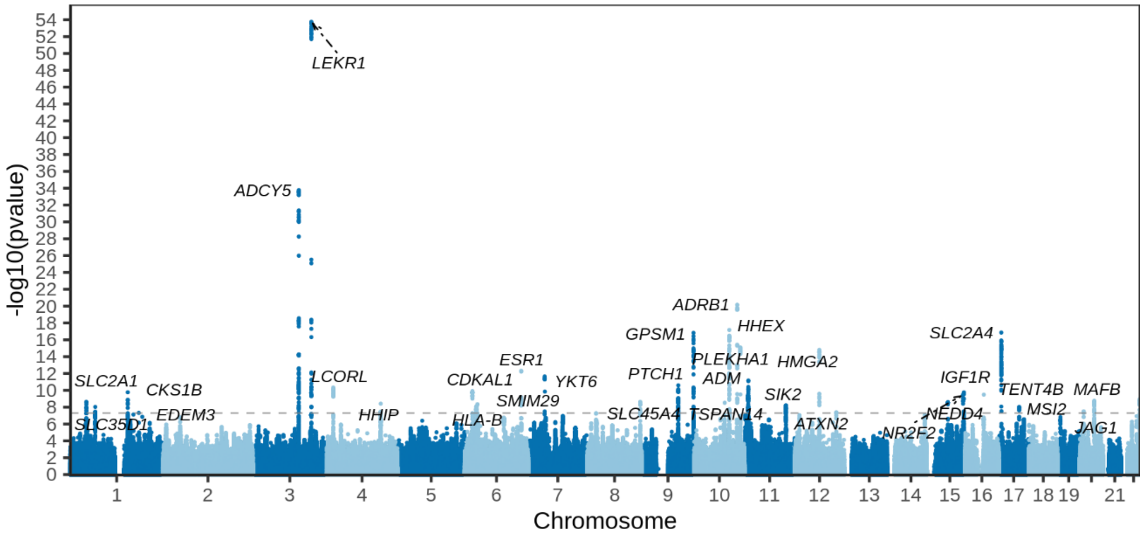

Supplementary Figure 4. QQ-plots and Manhattan plots of birth weight (z-score) genome-wide association studies on the foetal genome. The QQ-plots shown for a) parity = zero, b) parity > zero, and c) the whole study population include the lambda genomic control and demonstrate little to no test statistic inflation. In the Miami plot for d) first pregnancy (parity = zero, top, green) and later pregnancies (parity > zero, bottom, purple), and the Manhattan plot for e) the whole study population, the dashed line represents the genome-wide significant level (p-value = 5 x 10^{-8}). The x-axis shows the chromosome position and y-axis the two-sided p value of the GWAS. Variants reaching the significance threshold have the nearest protein-coding gene displayed.

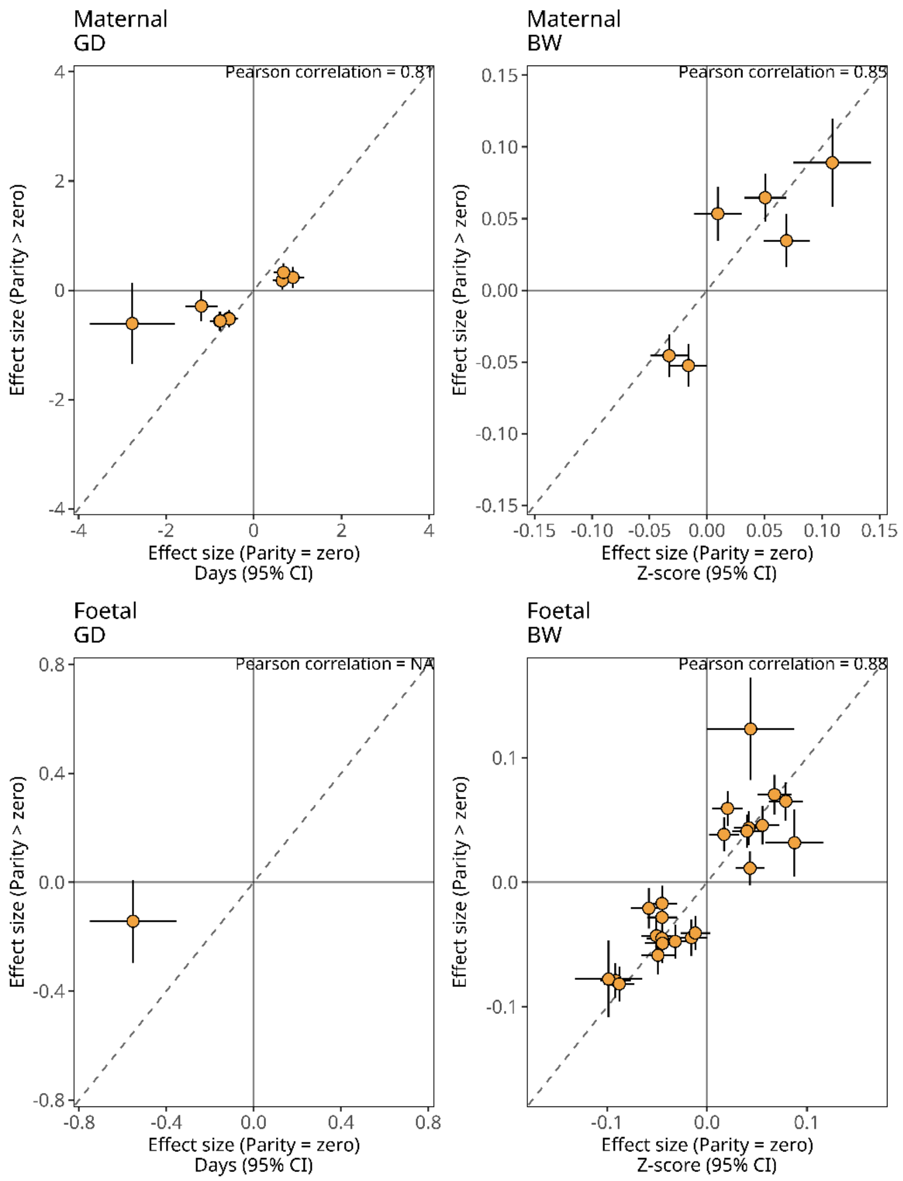

Supplementary Figure 5. Effect sizes (differences in days or z-score) for gestational duration (GD) and birth weight (BW) in the maternal (top) and foetal (bottom) parity stratified genome-wide association studies. The figure only includes variants that reached genome-wide significance in either the first pregnancy GWAS or later pregnancy GWAS. The dashed line is the identity line. The error bars are the 95% confidence interval. The Pearson correlation between the parity-stratified genetic effects for each specific outcome is displayed at the top of each subfigure.

a) QQ-plot for the maternal genome-wide association study between first and later-born (as a case-control study)

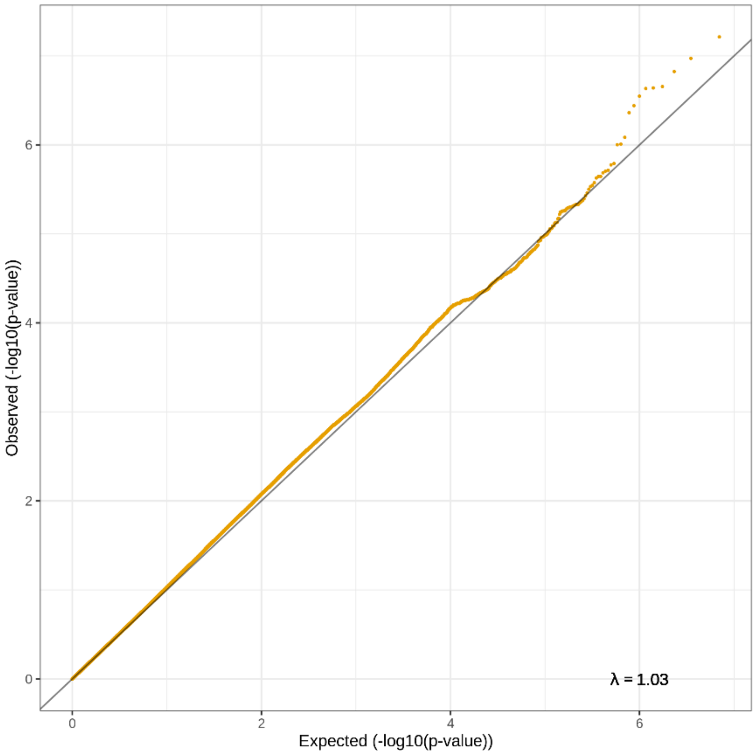

b) QQ-plot for the foetal genome-wide association study between first and later-born (as a case-control study)

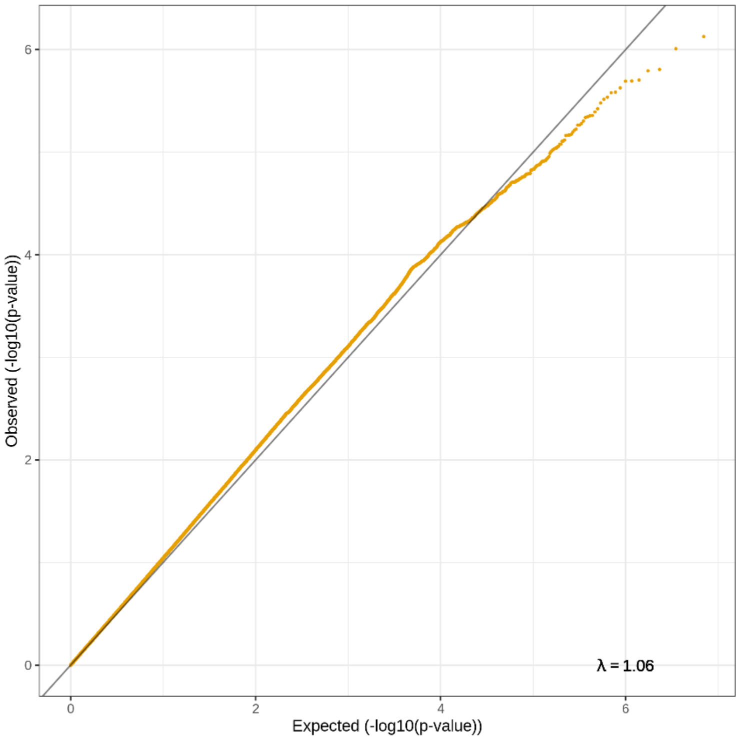

c) Manhattan plot for the maternal genome-wide association study between first and later-born (as a case-control study)

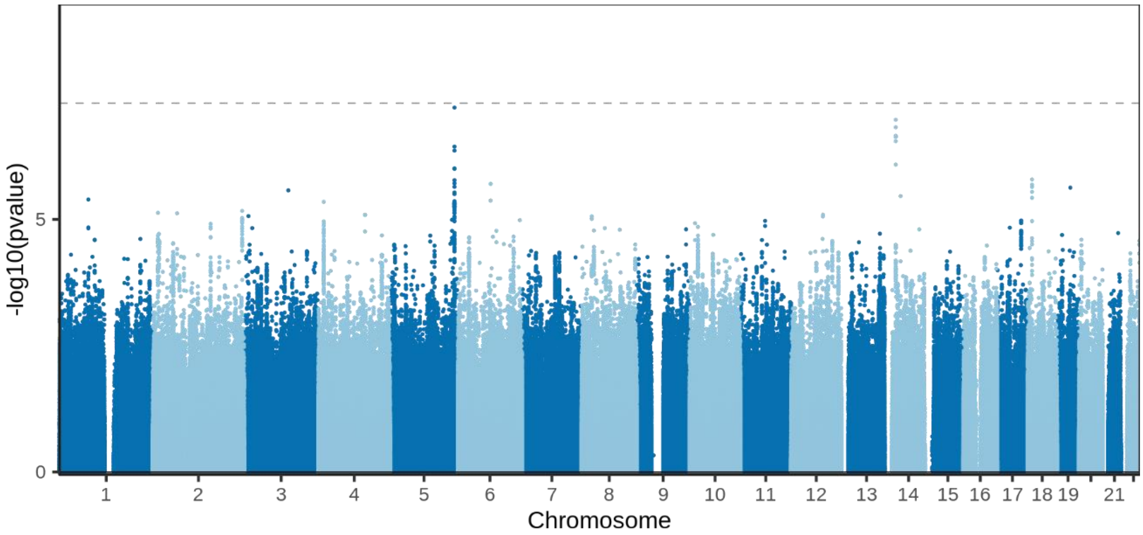

d) Manhattan plot for the foetal genome-wide association study between first and later-born (as a case-control study)

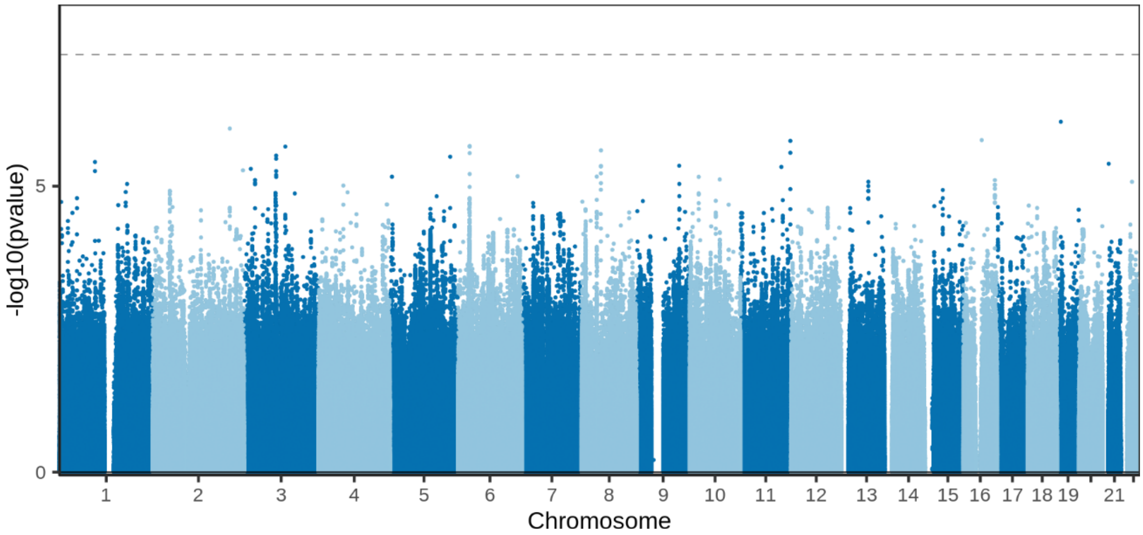

Supplementary Figure 6. QQ-plots and Manhattan plots for the genome-wide association study between first and later-born (as a case-control study). The QQ-plots shown in a) for the maternal genome and b) for the foetal genome include the lambda genomic control and demonstrate little to no test statistic inflation. In the Manhattan plots for c) the maternal genome and d) the foetal genome, the dashed line represents the genome-wide significant level (p-value = 5×10^{-8}). The x-axis shows the chromosome position and y-axis the two-sided p value of the GWAS. Variants reaching the significance threshold have the nearest protein-coding gene displayed.

a)

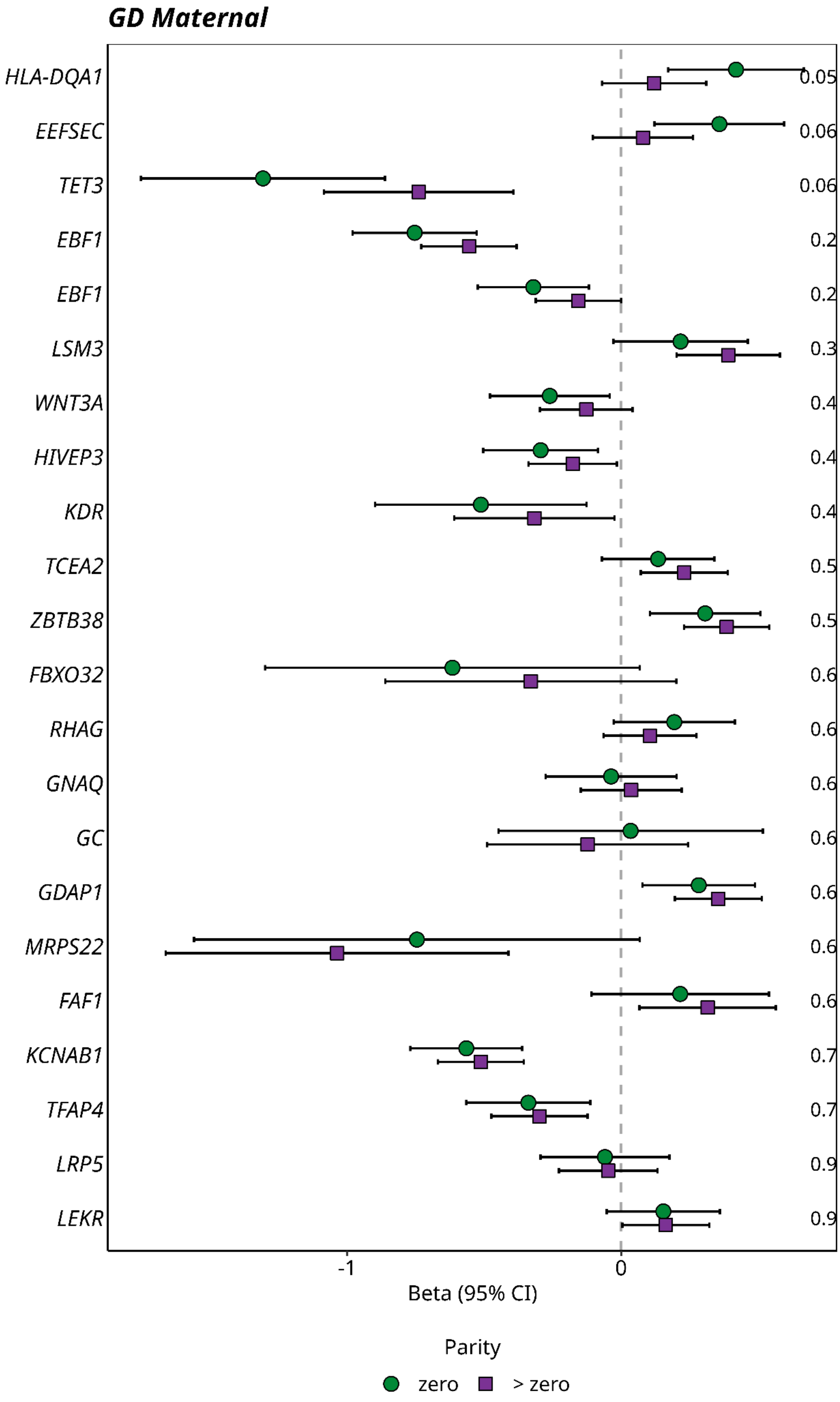
GD Maternal
HLA-DQA1
EEFSEC
TET3
EBF1
EBF1
LSM3
WNT3A
HIVEP3
KDR
TCEA2
ZBTB38
FBXO32
RHAG
GNAQ
GC
GDAP1
MRPS22
FAF1
KCNAB1
TFAP4
LRP5
LEKR
0.05
0.06
0.06
0.2
0.2
0.3
0.4
0.4
0.4
0.5
0.5
0.6
0.6
0.6
0.6
0.6
0.6
0.6
0.7
0.7
0.9
0.9
-1
0
Beta (95% CI)
Parity
zero
> zero

b)

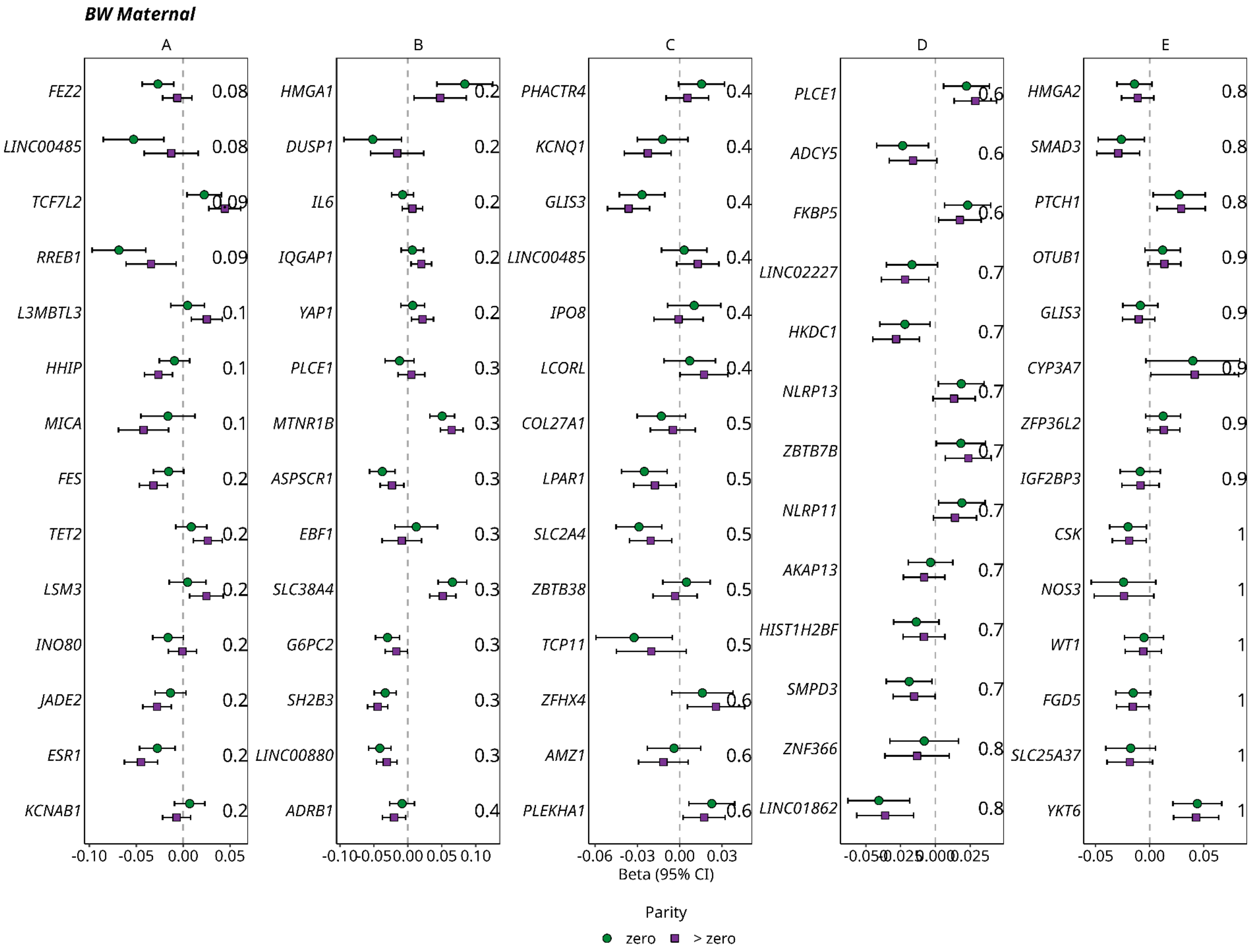
BW Maternal
A
FEZ2 0.08
LINC00485 0.08
TCF7L2 0.09
RREB1 0.09
L3MBTL3 0.1
HHIP 0.1
MICA 0.1
FES 0.2
TET2 0.2
LSM3 0.2
INO80 0.2
JADE2 0.2
ESR1 0.2
KCNAB1 0.2
-0.10 -0.05 0.00 0.05
B
HMGA1 0.2
DUSP1 0.2
IL6 0.2
IQGAP1 0.2
YAP1 0.2
PLCE1 0.3
MTNR1B 0.3
ASPSCR1 0.3
EBF1 0.3
SLC38A4 0.3
G6PC2 0.3
SH2B3 0.3
LINC00880 0.3
ADRB1 0.4
-0.10 -0.05 0.00 0.05 0.10
C
PHACTR4 0.4
KCNQ1 0.4
GLIS3 0.4
LINC00485 0.4
IPO8 0.4
LCORL 0.4
COL27A1 0.5
LPAR1 0.5
SLC2A4 0.5
ZBTB38 0.5
TCP11 0.5
ZFHX4 0.6
AMZ1 0.6
PLEKHA1 0.6
-0.06 -0.03 0.00 0.03
Beta (95% CI)
D
PLCE1 0.6
ADCY5 0.6
FKBP5 0.6
LINC02227 0.7
HKDC1 0.7
NLRP13 0.7
ZBTB7B 0.7
NLRP11 0.7
AKAP13 0.7
HIST1H2BF 0.7
SMPD3 0.7
ZNF366 0.8
LINC01862 0.8
-0.050 -0.025 0.000 0.025
E
HMGA2 0.8
SMAD3 0.8
PTCH1 0.8
OTUB1 0.9
GLIS3 0.9
CYP3A7 0.9
ZFP36L2 0.9
IGF2BP3 0.9
CSK 1
NOS3 1
WT1 1
FGD5 1
SLC25A37 1
YKT6 1
-0.05 0.00 0.05
Parity
zero
> zero

c)

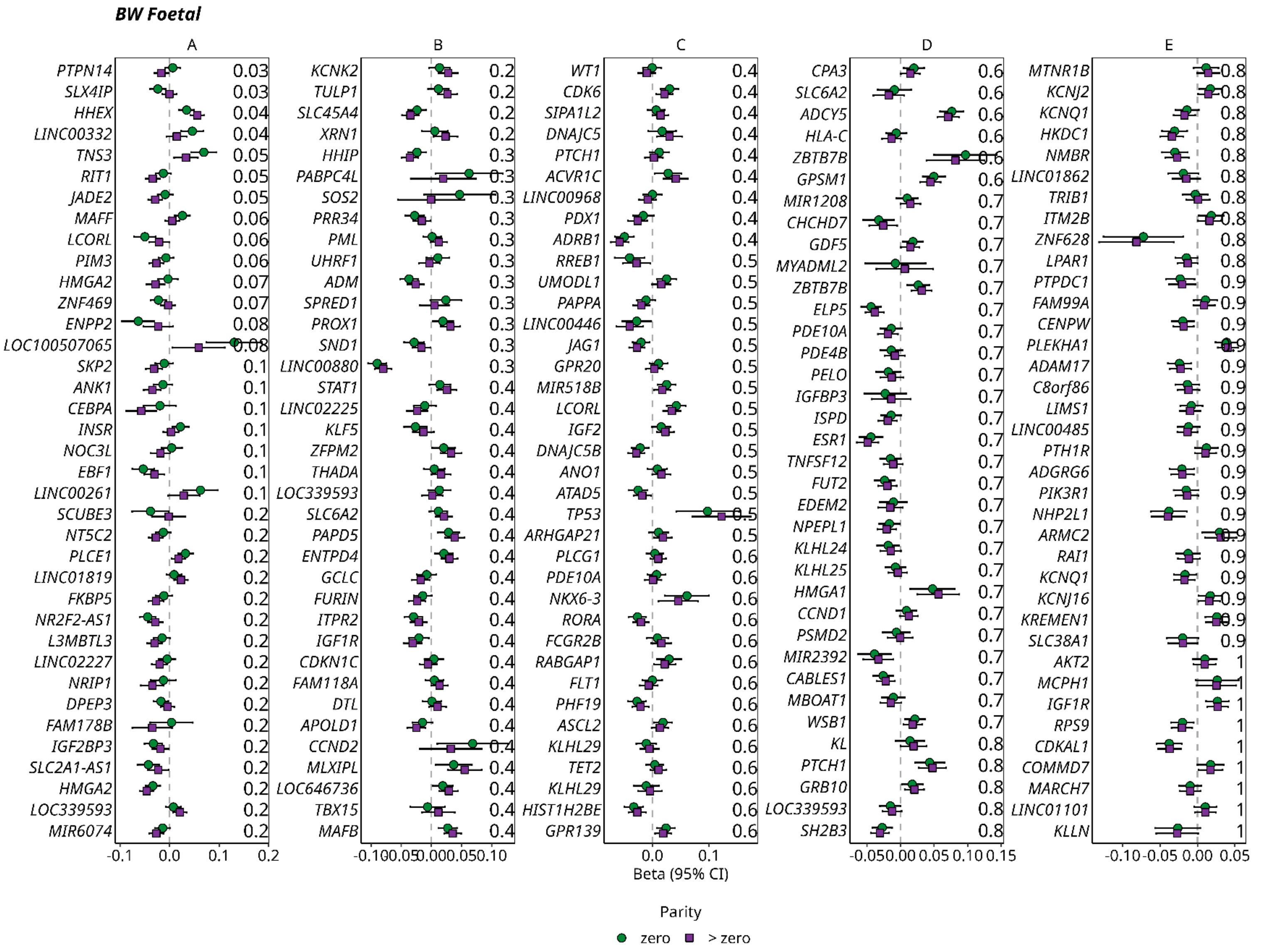
BW Foetal
A
PTPN14 0.03
SLX4IP 0.03
HHEX 0.04
LINC00332 0.04
TNS3 0.05
RIT1 0.05
JADE2 0.05
MAFF 0.06
LCORL 0.06
PIM3 0.06
HMGA2 0.07
ZNF469 0.07
ENPP2 0.08
LOC100507065 0.08
SKP2 0.1
ANK1 0.1
CEBPA 0.1
INSR 0.1
NOC3L 0.1
EBF1 0.1
LINC00261 0.1
SCUBE3 0.2
NT5C2 0.2
PLCE1 0.2
LINC01819 0.2
FKBP5 0.2
NR2F2-AS1 0.2
L3MBTL3 0.2
LINC02227 0.2
NRIP1 0.2
DPEP3 0.2
FAM178B 0.2
IGF2BP3 0.2
SLC2A1-AS1 0.2
HMGA2 0.2
LOC339593 0.2
MIR6074 0.2
-0.1 0.0 0.1 0.2
B
KCNK2 0.2
TULP1 0.2
SLC45A4 0.2
XRN1 0.2
HHIP 0.3
PABPC4L 0.3
SOS2 0.3
PRR34 0.3
PML 0.3
UHRF1 0.3
ADM 0.3
SPRED1 0.3
PROX1 0.3
SND1 0.3
LINC00880 0.3
STAT1 0.4
LINC02225 0.4
KLF5 0.4
ZFPM2 0.4
THADA 0.4
LOC339593 0.4
SLC6A2 0.4
PAPD5 0.4
ENTPD4 0.4
GCLC 0.4
FURIN 0.4
ITPR2 0.4
IGF1R 0.4
CDKN1C 0.4
FAM118A 0.4
DTL 0.4
APOLD1 0.4
CCND2 0.4
MLXIPL 0.4
LOC646736 0.4
TBX15 0.4
MAFB 0.4
-0.10 -0.05 0.00 0.05 0.10
C
WT1 0.4
CDK6 0.4
SIPA1L2 0.4
DNAJC5 0.4
PTCH1 0.4
ACVR1C 0.4
LINC00968 0.4
PDX1 0.4
ADRB1 0.4
RREB1 0.5
UMODL1 0.5
PAPPA 0.5
LINC00446 0.5
JAG1 0.5
GPR20 0.5
MIR518B 0.5
LCORL 0.5
IGF2 0.5
DNAJC5B 0.5
ANO1 0.5
ATAD5 0.5
TP53 0.5
ARHGAP21 0.5
PLCG1 0.6
PDE10A 0.6
NKX6-3 0.6
RORA 0.6
FCGR2B 0.6
RABGAP1 0.6
FLT1 0.6
PHF19 0.6
ASCL2 0.6
KLHL29 0.6
TET2 0.6
KLHL29 0.6
HIST1H2BE 0.6
GPR139 0.6
0.0 0.1
Beta (95% CI)
D
CPA3 0.6
SLC6A2 0.6
ADCY5 0.6
HLA-C 0.6
ZBTB7B 0.6
GPSM1 0.6
MIR1208 0.7
CHCHD7 0.7
GDF5 0.7
MYADML2 0.7
ZBTB7B 0.7
ELP5 0.7
PDE10A 0.7
PDE4B 0.7
PELO 0.7
IGFBP3 0.7
ISPD 0.7
ESR1 0.7
TNFSF12 0.7
FUT2 0.7
EDEM2 0.7
NPEPL1 0.7
KLHL24 0.7
KLHL25 0.7
HMGA1 0.7
CCND1 0.7
PSMD2 0.7
MIR2392 0.7
CABLES1 0.7
MBOAT1 0.7
WSB1 0.7
KL 0.8
PTCH1 0.8
GRB10 0.8
LOC339593 0.8
SH2B3 0.8
-0.05 0.00 0.05 0.10 0.15
E
MTNR1B 0.8
KCNJ2 0.8
KCNQ1 0.8
HKDC1 0.8
NMBR 0.8
LINC01862 0.8
TRIB1 0.8
ITM2B 0.8
ZNF628 0.8
LPAR1 0.8
PTPDC1 0.9
FAM99A 0.9
CENPW 0.9
PLEKHA1 0.9
ADAM17 0.9
C8orf86 0.9
LIMS1 0.9
LINC00485 0.9
PTH1R 0.9
ADGRG6 0.9
PIK3R1 0.9
NHP2L1 0.9
ARMC2 0.9
RAI1 0.9
KCNQ1 0.9
KCNJ16 0.9
KREMEN1 0.9
SLC38A1 0.9
AKT2 1
MCPH1 1
IGF1R 1
RPS9 1
CDKAL1 1
COMMD7 1
MARCH7 1
LINC01101 1
KLLN 1
-0.10 -0.05 0.00 0.05
Parity
zero
> zero

Supplementary Figure 7. Parity stratified effect sizes (n ~ 25,000 mothers/ children) for a) gestational duration (GD) maternal genome, b) birth weight (BW) maternal genome and c) BW foetal genome. The green circle are the effect sizes in first pregnancy and the purple square are the effect sizes in the later pregnancies. The error bars are the 95% confidence interval. Variants are displayed as the nearest protein-coding gene on the y-axis. The interaction p-value for each variant is displayed to the right. Only variants previously associated with the specific outcome in (2-7) were included. The five variants with the lowest interaction p-value are illustrated in Figure 3.
